# High-plex immunofluorescence imaging and traditional histology of the same tissue section for discovering image-based biomarkers

**DOI:** 10.1038/s43018-023-00576-1

**Published:** 2023-06-22

**Authors:** Jia-Ren Lin, Yu-An Chen, Daniel Campton, Jeremy Cooper, Shannon Coy, Clarence Yapp, Juliann B. Tefft, Erin McCarty, Keith L. Ligon, Scott J. Rodig, Steven Reese, Tad George, Sandro Santagata, Peter K. Sorger

**Affiliations:** 1 Laboratory of Systems Pharmacology, Department of Systems Biology, Harvard Medical School, Boston, MA USA; 2 Ludwig Center at Harvard, Harvard Medical School, Boston, MA USA; 3 RareCyte, Inc., Seattle, WA USA; 4 Department of Pathology, Brigham and Women’s Hospital, Harvard Medical School, Boston, MA USA

**Keywords:** Colon cancer, Machine learning, Cancer imaging, Tumour biomarkers, Cancer

## Abstract

Precision medicine is critically dependent on better methods for diagnosing and staging disease and predicting drug response. Histopathology using hematoxylin and eosin (H&E)-stained tissue (not genomics) remains the primary diagnostic method in cancer. Recently developed highly multiplexed tissue imaging methods promise to enhance research studies and clinical practice with precise, spatially resolved single-cell data. Here, we describe the ‘Orion’ platform for collecting H&E and high-plex immunofluorescence images from the same cells in a whole-slide format suitable for diagnosis. Using a retrospective cohort of 74 colorectal cancer resections, we show that immunofluorescence and H&E images provide human experts and machine learning algorithms with complementary information that can be used to generate interpretable, multiplexed image-based models predictive of progression-free survival. Combining models of immune infiltration and tumor-intrinsic features achieves a 10- to 20-fold discrimination between rapid and slow (or no) progression, demonstrating the ability of multimodal tissue imaging to generate high-performance biomarkers.

## Abstract (web summary)

Sorger and colleagues present Orion, a technique to analyze H&E histology and multiplex immunofluorescence imaging data from the same cells, which they apply to human colorectal cancer samples to identify spatial biomarkers of disease progression.

## Main

The microanatomy of fixed and stained tissues has been studied using light microscopy for over two centuries^1,2^, and histopathology review of hematoxylin and eosin (H&E)-stained tissue sections, complemented by immunohistochemistry (IHC) and exome sequencing, remains the primary approach for diagnosing and managing many diseases, particularly cancer^3^. More recently, machine learning and artificial intelligence (ML/AI) approaches have been developed to automatically extract information from H&E images^4^, leading to progress in computer-assisted diagnosis^5^. However, H&E and IHC images generally lack the precision and depth of molecular information needed to optimally predict outcomes, guide the selection of targeted therapies and enable research into mechanisms of disease^6^.

The transition of histopathology to digital approaches^7^ is concurrent with the introduction, in research settings, of methods for obtaining 10- to 100-plex imaging data from tissues (for example, multiplex immunofluorescence (MxIF), cyclic immunofluorescence (CyCIF), COdetection by inDEXing (CODEX), iterative indirect immunofluorescence imaging (4i), multiplex immunohistochemistry (mIHC), multiplexed ion beam imaging (MIBI), iterative bleaching extends multiplexity (IBEX) and imaging mass cytometry (IMC))^8–14^. Such approaches combine subcellular-resolution morphological analysis with spatially resolved molecular data and are ideal complements to dissociative single-cell methods, such as single-cell RNA sequencing. Methods compatible with the type of specimens universally acquired for diagnostic purposes (formaldehyde-fixed and paraffin-embedded (FFPE) specimens) also make it possible to tap into large archives of human biopsy and resection specimens. Many high-plex imaging studies performed to date on human cohorts involve tissue microarrays (TMAs; arrays of many 0.6- to 1-mm diameter specimens on a single slide) or the small fields of view characteristic of mass spectrometry-based imaging^8,10^, but whole-slide imaging (WSI) is required for clinical research and diagnosis, both to achieve sufficient statistical power^15^ and as a Food and Drug Administration requirement^16^.

During histopathology review of H&E images, a human expert draws on implicit and explicit knowledge about the abundances and morphologies of cellular and acellular structures prognostic of disease or predictive of drug response. This prior knowledge, summarized in resources such as the American Joint Committee on Cancer’s staging manual^17^, is based on thousands of clinical research papers and numerous clinical trials. By contrast, research using highly multiplexed imaging relies primarily on spatial statistics, which recognizes recurrent patterns in a data-driven manner^8,10^ but has not yet been subjected to rigorous validation in a clinical setting. An opportunity therefore exists to combine deep knowledge of tissue anatomy (acquired from H&E images)^18^ with newly acquired insights into single cell types and states. We reasoned that an ideal instrument for achieving this would perform WSI^19^, have sufficient plex and resolution to distinguish tumor, immune and stromal cell types and enable reliable and efficient data acquisition with minimal human intervention. In current practice, combining immunofluorescence (IF) and H&E imaging requires the use of different tissue sections^20^. However, collection of same-cell multimodal images would enable one-to-one comparison of cell morphologies and molecular properties and also facilitate integration with ML/AI approaches being developed for H&E data^21^.

The relative complexity of existing highly multiplexed imaging assays has slowed their adoption in the clinic; the current standard in clinical research is five- to six-plex imaging using a PerkinElmer Vectra Polaris (now Akoya PhenoImager HT)^22^. However, a first-principles analysis suggests that a minimum of 16–20 molecular (IF) channels are required for tumor profiling (Supplementary Table 1), 10–12 are required to subtype major immune cell types, 2–3 are required to detect and subtype tumor cells and states, 2–4 are required to identify relevant tissue structures and 1–3 are required to examine tumor cells states or therapeutic mechanisms, plus a nuclear stain to locate cell nuclei. Achieving this would benefit from acquisition of many fluorescent channels in parallel (one-shot imaging) rather than the sequential process developed by Gerdes et al.^9^ and subsequently extended by our group^14^ and others^23^.

In this paper, we describe the development of an approach to one-shot, whole-slide, 16- to 18-channel IF imaging, followed by H&E staining and imaging of the same cells. We compare the performance of the ‘Orion’ approach and a commercial-grade instrument that implements it with established IHC and cyclic data acquisition by CyCIF^24^. Using both human inspection and ML on multimodal Orion images, we demonstrate beneficial transfer of information from H&E images to high-plex IF data (for example, to distinguish normal tissue from a tumor) and also the other way around (for example, to subtype immune cells that are indistinguishable in H&E data). In a proof-of-principle study, we use two independent 30- to 40-participant human colorectal cancer (CRC) cohorts (*N* = 74 participants total) to identify spatial biomarkers prognostic of tumor progression with hazard ratios (HRs) of 0.05 to 0.15 (that is, up to a 20-to-1 discrimination of rapid versus slow progression). Thus, the Orion method makes multimodal data accessible and compatible with cohort studies and eventual use in diagnosis.

## Results

### Constructing and testing the Orion platform

We investigated multiple approaches for achieving one-shot high-plex IF followed by H&E imaging of the same tissue section. Overlap in the excitation and emission spectra of the most widely used fluorophores limits the number of separable fluorescence channels (typically five to six) that can be accommodated within the wavelengths useful for antibody labeling (~350 to 800 nm). This can be overcome using tuned emission and excitation filters and spectral deconvolution (for example, six to ten channels)^25^ or by dispersing emitted light using a diffraction grating and then performing linear unmixing^26,27^. However, unmixing complex spectra has historically resulted in a substantial reduction in sensitivity and has not been widely implemented. Simultaneous high-plex imaging of tissue specimens therefore requires innovation in the optical platform as well as careful selection of fluorophores.

**Fig. 1:**
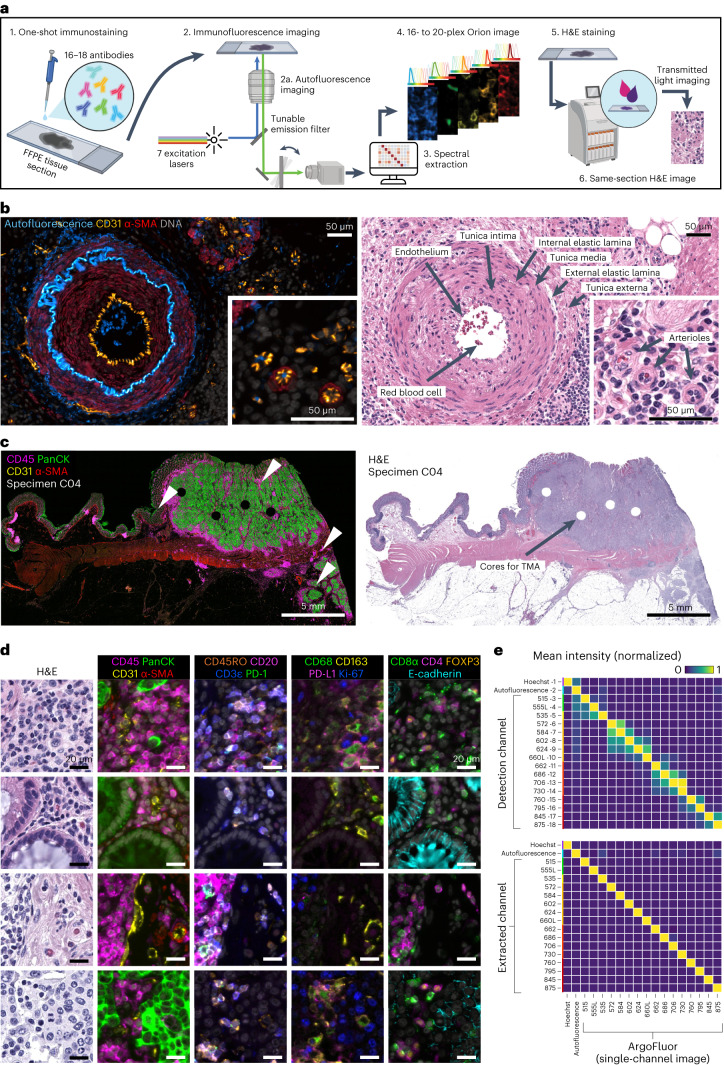
Same-section IF and H&E using the Orion platform. **a**, Schematic of one-shot 16- to 20-channel multiplexed IF imaging with the Orion method followed by H&E staining of the same section using an automated slide stainer and scanning of the H&E-stained slide in transillumination (brightfield) mode. This method of discriminating the emission spectra of fluorophores is repeated using seven excitation lasers spaced across the spectrum (see Extended Data Fig. 1b and Methods). Using polychroic mirrors and tunable optical filters, emission spectra are extracted to discriminate up to 20 channels, including signal from fluorophore-labeled antibodies (15–19 in most experiments), the nuclear stain Hoechst 33342 and tissue-intrinsic autofluorescence (figure created with BioRender.com). **b**, Left, Orion multiplexed IF image showing CD31, α-SMA, Hoechst (DNA) and signal from the tissue autofluorescence channel; this image highlights an artery outside of the tumor region with red blood cells in the vessel lumen and elastic fibers in the internal and external elastic lamina of the vessel wall, numerous smaller vessels (arterioles) and stromal collagen fibers (the inset displays arterioles). Right, images of the H&E staining from the same tissue section (histologic landmarks are indicated). Images are from a single representative specimen (C18). **c**, Orion multiplexed IF image (showing CD45, pan-cytokeratin (PanCK), CD31 and α-SMA) from a whole-tissue FFPE section and matched H&E from the same section. Holes in the images are regions of tissue (‘cores’) removed in the construction of TMAs. Images are from a single representative specimen (C04). **d**, Zoom-in views of the regions indicated by arrowheads in **c**; marker combinations are indicated. The images are from a single representative specimen (C04). **e**, Intensities of fluorochromes (columns in heat maps) in each Orion channel (rows in heat maps) before (top) and after (bottom) spectral extraction. The extraction matrix was determined from control samples scanned using the same acquisition settings that were used for the full panel. The control samples included unstained lung tissue (for the autofluorescence channel), tonsil tissue stained with Hoechst and tonsil tissue stained in single plex with ArgoFluor conjugates used in the panel (for the biomarker channels). The values in each column were normalized to the maximum value in the column. Data were derived from a single pool (*N* = 1) of control beads. Source data

**Extended Data Fig. 1:**
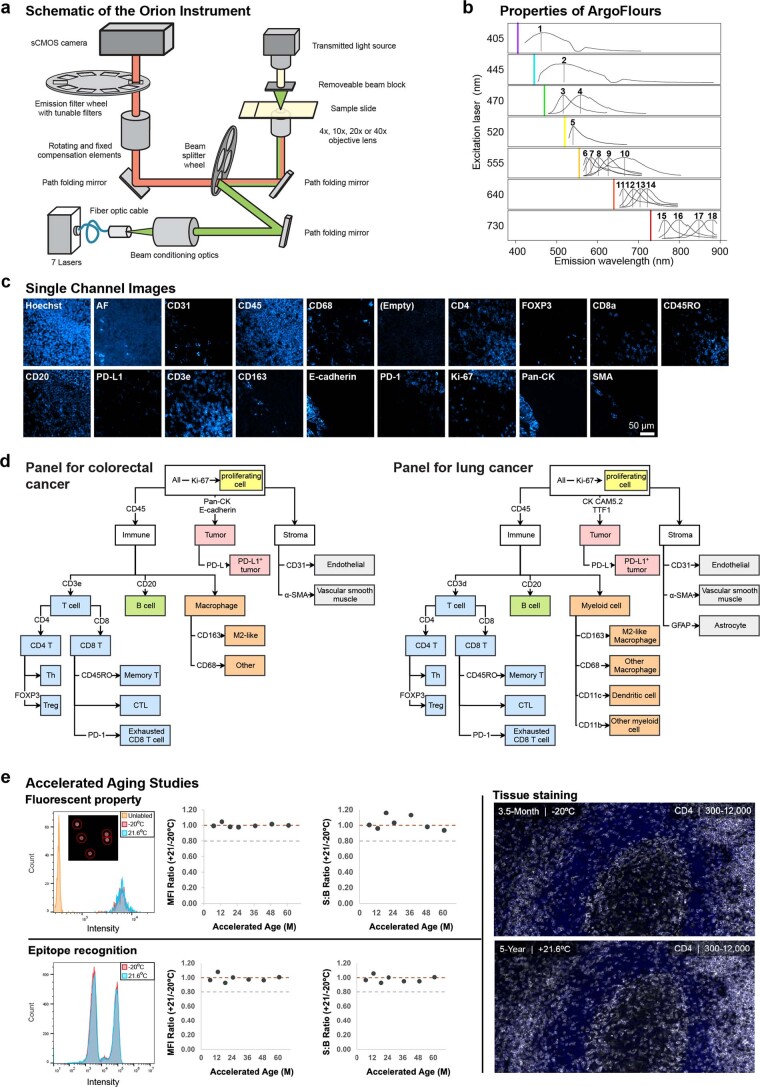
Features of the fluorophores, signal extraction, antibodies, cell type calling, and instrumentation used in the Orion™ Method. **a**, Schematic of the Orion optical system. The Orion imaging system has fluorescence and brightfield imaging modes. *Fluorescence imaging*: A 7-color, class 1 laser was used to illuminate a sample slide. Emission light from the sample is redirected through a tunable emission filter prior to collection by a sCMOS camera. *Brightfield imaging*: The Orion system utilizes LED transillumination of the microscope slide sample. Transmitted light follows the same path as the fluorescence emission, with the exception that a window is used instead of an emission filter. **b**, Emission spectra of the ArgoFluor dyes with overlaid filter profiles. Each row shows fluorophores excited using the same laser (denoted by the colored vertical line). See Supplementary Table 2 for excitation laser wavelengths and fluorophores in channel 1 through 18. The 405-laser data was collected from tonsil tissue stained with Hoechst 33342; 445-laser data from unstained lung tissue. All other data was collected from single color Ig-capture beads (generated by incubation with antibodies conjugated to the indicated ArgoFluor dye). Per sample, data was collected in multiple Orion channels, spanning a wide range of wavelengths (in 2 nm center wavelength increments). **c**, Single channel images of FFPE tonsil section stained, imaged, and processed with Orion showing distinct spatial patterns and minimal channel crosstalk. **d**, Cell type calling dendrograms for Orion image analysis for colorectal cancer (left) and lung cancer (right). **e**, Stability of ArgoFluor 572 conjugated anti-CD4 antibody. Reagents were stored at an accelerated aging condition (21.6 °C) or the recommended condition (−20 °C). Storage for 3.5 months at 21.6 °C is equivalent to 5 years at −20 °C based on the Arrhenius equation. *Fluorochrome property*: The intensity of Ig-capture beads incubated with (signal) or without (background) antibody was measured from Orion images. The histogram overlay shows the intensity distribution for beads that were unlabeled or incubated with antibody and stored for 3.5 months at −20 °C or 21.6 °C. The mean fluorescence intensity (MFI) and the MFI signal-to-background (S:B) ratios were obtained across 7 time points. *Epitope recognition*: Human peripheral blood mononuclear cells (PBMC) were stained with accelerated-aged or real-time-aged ArgoFluor 572 conjugated anti-CD4 antibody and analyzed using flow cytometry. The MFI was obtained for the positive and negative populations to derive S:B ratios. *Tissue staining*: Orion images of serial sections from FFPE tonsil stained with real-time aged (top) and accelerated-aged (bottom) antibodies. These methods demonstrate equivalent performance for both storage conditions in the three assays. Source data

With support from an NCI SBIR grant, a commercial-grade Orion instrument was developed that uses seven lasers (Fig. 1a and Extended Data Fig. 1a) to illuminate the sample and collect emitted light with ×4 to ×40 objective lenses (0.2 NA to 0.95 NA; Orion data in this paper were collected with a 20x/0.75 NA objective), followed by multiple tunable optical filters that use a non-orthogonal angle of incidence on thin-film interference filters to shift the emission bandpass^28^. These filters have 90–95% transmission efficiency and enable collection of 10- to 15-nm bandpass channels with 1-nm center wavelength (CWL) tuning selectivity over a wide range of wavelengths (425 to 895 nm). Narrow bandpass emission channels improve specificity but substantially reduce signal strength; we overcame this problem by using excitation lasers that are approximately tenfold brighter than conventional LED illuminators and by using a sensitive scientific CMOS detector (camera). Raw image files were then processed computationally to correct for system aberrations, such as geometric distortions and camera non-linearity^29^, followed by spectral extraction to remove cross-talk and isolate individual fluorophore signals (and thus the antibodies to which they were conjugated). The features of single cells and regions of tissue were then computed using MCMICRO software^30^.

We tested >100 chemical fluorophores from different sources and identified 18 ArgoFluors that were compatible with spectral extraction enabled by discrete sampling. Key criteria were (1) emission in the 500- to 875-nm range, (2) high quantum efficiency, (3) good photostability and (4) compatibility with each other in high-plex panels (Extended Data Fig. 1b and Supplementary Tables 1 and 2). ArgoFluor dyes were covalently coupled to commercial antibodies directed to lineage markers of immune (for example, CD4, CD8 and CD68), epithelial (cytokeratin and E-cadherin) and endothelial (CD31) cells as well as immune checkpoint regulators (PD-1 and PD-L1) and cell state markers (Ki-67) to generate panels suitable for studying the microenvironment and architecture of epithelial tumors and adjacent normal tissue (Extended Data Fig. 1c,d). An accelerated aging test demonstrated excellent reagent stability, estimated to be >5 years at −20 °C storage (Extended Data Fig. 1e).

**Extended Data Fig. 2:**
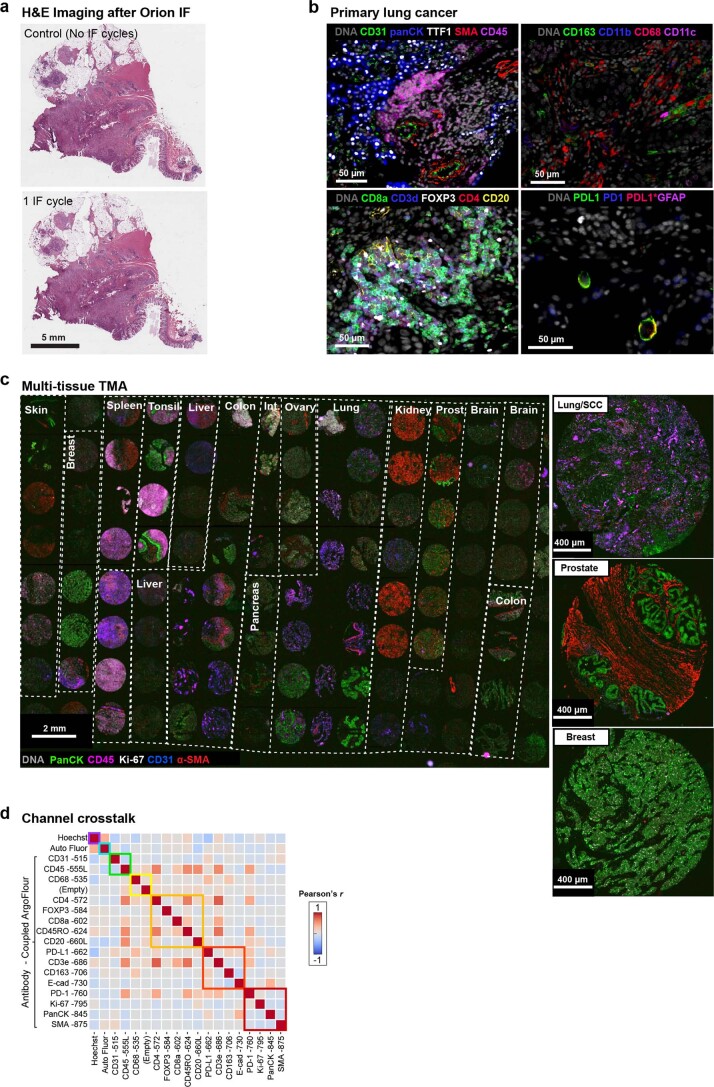
Orion imaging of different cancer types (colorectal and lung) and assessment of channel crosstalk. **a**. Images of H&E**-**stained sections of colorectal cancer performed before IF imaging (no IF cycles) and after one cycle of IF imaging (1 IF cycle) showing excellent preservation of staining intensity and morphology. Scalebars 5 mm. Images from one sample. **b**, Representative images of 20-plex Orion panel from a primary lung adenocarcinoma sample. Note: two PD-L1 antibodies were used, PD-L1 (green) is E1L3N clone from Cell Signaling and PD-L1*(red) is EPR19759 from Abcam. Scalebars 50 µm. Images from one sample. **c**, 16-plex (18 channel) Orion image from a tissue microarray (TMA) containing normal and diseased human tissues including inflammatory and neoplastic diseases (Examples highlighted are lung squamous cell carcinoma (SCC), prostate adenocarcinoma, and breast ductal carcinoma); DNA, pan-cytokeratin, Ki-67, α-SMA, CD45 and CD31 are displayed. Scalebars 2 mm and 400 µm, as indicated. Images from one TMA containing 123 patient samples. **d**, Validation of minimal channel crosstalk in 18-plex tonsil image after spectral extraction. Pearson’s correlation coefficients between all channel pairs were calculated using the paired pixel intensities. Square boxes with colored borders denote excitation lasers. High correlation coefficients were only found in channel pairs that contains target markers that are in close proximity. Data was derived from a selected frame of (*N* =1) image. Source data

Because eosin fluoresces strongly at 530–620 nm, it proved impractical to perform H&E staining before IF (although alternatives to H&E compatible with IF have been described)^31^. However, we found that H&E staining could be performed using industry-standard slide stainers after one or a small number of IF cycles. No established methods exist for evaluating the quality of these or other digital H&E images^32^, and comparison is complicated by variation in color intensity across platforms. We therefore acquired H&E images using an Aperio GT450 slide scanner (Leica Biosystems), which is a gold standard for diagnostic applications^33^, rather than the integrated Orion brightfield mode. Four practicing pathologists were shown images of tissue sections that had been subjected to one or more IF staining cycles followed by fluorophore bleaching and asked were whether they could distinguish these images from serial section controls that had been stained with H&E in the standard manner in a clinical facility (Fig. 1a). They found the two sets of images to be indistinguishable and ‘diagnostic grade’ (Extended Data Fig. 2a). We conclude that good-quality post-Orion H&E imaging can be obtained, although further study of additional tissues will be required to fully assess whether they are generally adequate for use in diagnosis.

### Validating high-plex one-shot fluorescence imaging

To test the Orion approach, three types of data were collected: (1) whole-slide images of human tonsil, a standard tissue for antibody qualification, and human lung cancer, a particularly common cancer type; (2) images of a TMA that contains 30 different types of normal non-neoplastic disease as well as tumor samples from 18 tissues and (3) whole-slide images of 74 stage I–IV CRC resections obtained from the archives of the Brigham and Women’s Hospital (BWH) Pathology Department (these resections were split into two cohorts with 40 and 34 individuals each, respectively, as indicated in Supplementary Table 3). We tested and optimized the antibody panel on tonsil tissue and then applied it successfully to the lung cancer specimen (Extended Data Fig. 2b), TMA (Extended Data Fig. 2c and Supplementary Table 4) and CRC cohort. We also collected data from a dedicated autofluorescence channel (445-nm excitation/485-nm emission, CWL) to extract natural fluorescence from the IF channels, improve biomarker signal-to-noise ratio (SNR) and provide information on naturally fluorescent structures, such as connective tissues and components of blood vessels (Fig. 1b). In each case, we performed 18- to 20-plex imaging (16–18 antibody channels, autofluorescence and a nuclear stain) plus H&E. Exploratory studies suggest that it should be possible to add two to four additional antibody channels to the method following further optimization of fluorophores and optical systems (Methods).

Inspection of whole-slide images of lung, tonsil and CRC confirmed error-free imaging and stitching of 1,000 or more adjacent tiles (area of up to 35 × 20 mm; Fig. 1c), including bright in-focus staining of cellular and cellular substructures within each tile (Fig. 1d). To quantify the effectiveness of spectral extraction, we imaged serial sections of human tonsil tissue each stained with a single antibody conjugated to a different ArgoFluor and then recorded data in all channels. Under these conditions, cross-talk between adjacent channels averaged ~35%. Spectral extraction reduced this to <1% (Fig. 1e). As a result, when a tissue section was subjected to multiplexed antibody labeling, we observed correlated signals only for antibodies that stain targets colocalized on the same types of cells (for example, co-staining of T cell membranes with anti-CD3ε and anti-CD4; Extended Data Fig. 2d).

**Fig. 2:**
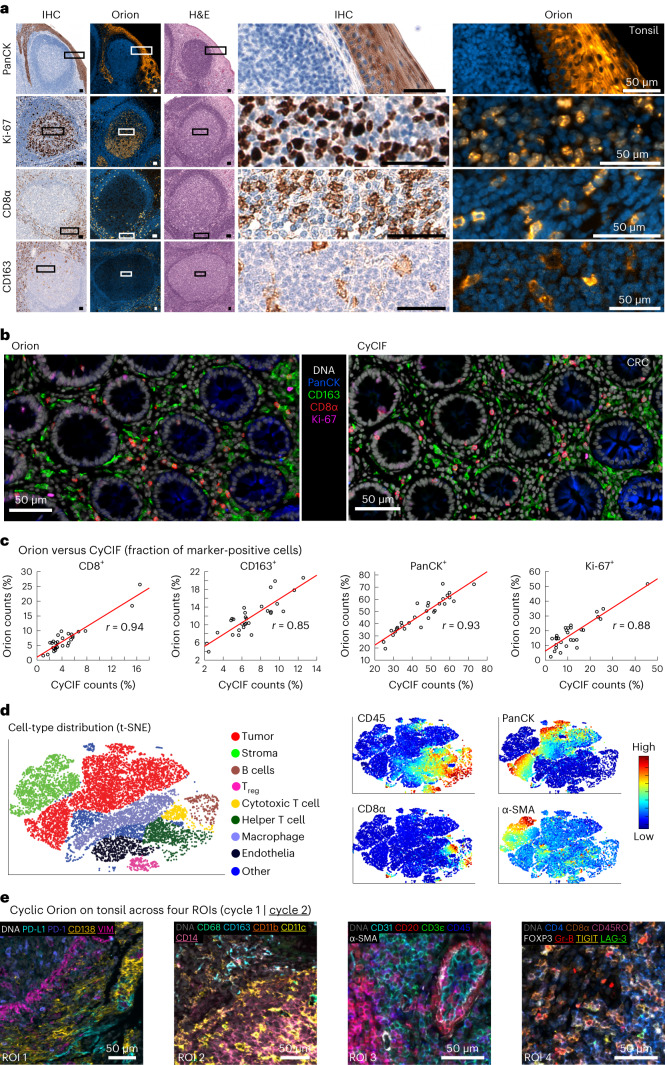
Qualifying the 16-plex single-shot Orion antibody panel. **a**, Panels of images from FFPE tonsil sections showing single-antibody IHC for pan-cytokeratin, Ki-67, CD8α, CD163 and the matching channels extracted from 16-plex Orion IF images (the H&E stain was performed on the same section as the Orion imaging). Each image is from one representative specimen. For IHC/H&E, four serial sections were used from the same tonsil tissue; one additional section from the same sample was used for Orion. **b**, Orion IF images and CyCIF images from neighboring sections of an FFPE colorectal adenocarcinoma. The CyCIF images were collected using 2 × 2 binning, while Orion images were obtained with no binning. **c**, Plots of the fraction of cells positive for the indicated markers from whole-slide Orion IF and CyCIF images acquired from neighboring sections. Pearson correlation coefficients are indicated. **d**, t-SNE plots of cells segmented from an Orion IF image of an FFPE CRC specimen (C01) with inferred cells types (left) and the fluorescence intensities of selected markers (CD45, pan-cytokeratin, CD8α and α-SMA; right) overlaid on the plots as heat maps. The plots show a random sample of 50,000 cells. **e**, Orion images showing antibodies imaged across two cycles. Twenty-three of 29 antibodies are displayed across four marker groups from four different regions of interest (labeled ROI 1–4). Markers from cycle 2 are underlined. The locations of the four ROIs in the whole-slide image are shown in Extended Data Fig. 5a. Images are from one FFPE tonsil specimen/section; VIM, vimentin; Gr-B, granzyme B. Source data

**Extended Data Fig. 3:**
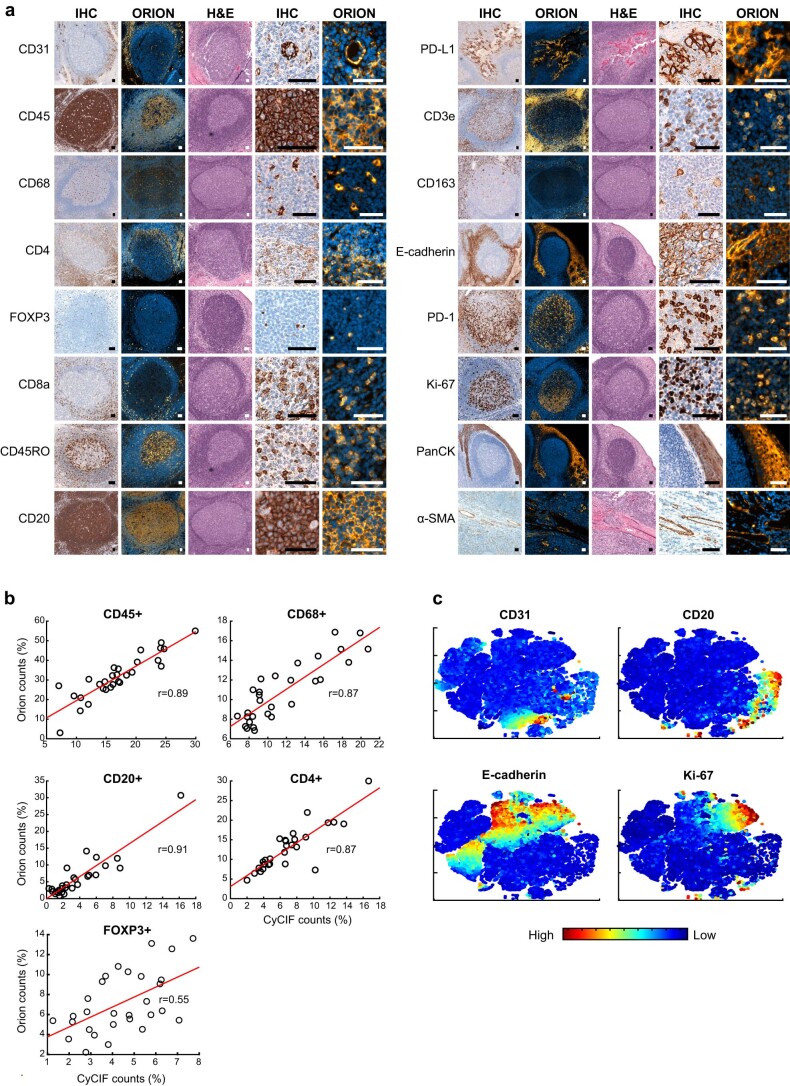
Qualifying 16-plex single-shot Orion antibody panel relative to immunohistochemistry and Cyclic Immunofluorescence (CyCIF). **a**, Panels of images from FFPE tonsil sections showing single-antibody immunohistochemistry (IHC) for the indicated markers and matching channels extracted from the 16-plex Orion immunofluorescence (IF) images (H&E stain was performed on the same section as the Orion imaging). Scalebars 50 µm. Images are from one representative tonsil specimen. **b**, Plots of the fraction of positive for the indicated markers (CD45, CD68, CD20, CD4, FOXP3) from whole-slide Orion IF and CyCIF images acquired from two adjacent sections of 29 FFPE colorectal cancer specimens. Pearson correlation coefficients are indicated. **c**, t-distributed stochastic neighbor embedding (t-SNE) plots of cells from Orion IF image. Log transformed marker intensities (CD31, CD20, E-cadherin, Ki-67) were used to color the dots in each panel. Fig. 2d contains tSNE plots for additional markers and the inferred cell types. Single cell data is from one representative specimen (C01). Source data

The staining patterns observed with ArgoFluor antibody conjugates were similar to those obtained by conventional IHC performed on the same specimen using the same antibody clones (as described in Du et al.^34^, one-to-one comparison of IF and IHC is not possible given fundamental differences in imaging modalities; Fig. 2a and Extended Data Fig. 3a). We also compared Orion data to data acquired from a serial tissue section using a well-established CyCIF method^14^. We found that the fractions of cells scoring positive for the same markers across the two methods were highly correlated (Fig. 2b,c shows four examples with *ρ* = 0.8 to 0.9) except when marker-positive cells were rare, and cell counts were subject to statistical fluctuation from one serial section to the next (for example, *ρ* = 0.55 for FOXP3 positivity; Extended Data Fig. 3b)^34^. Moreover, projections of high-dimensional Orion data using t-distributed stochastic neighbor embedding (t-SNE) revealed good marker separation and successfully resolved the anticipated populations of immune and tumor cell types (Fig. 2d and Extended Data Fig. 3c).

**Extended Data Fig. 4:**
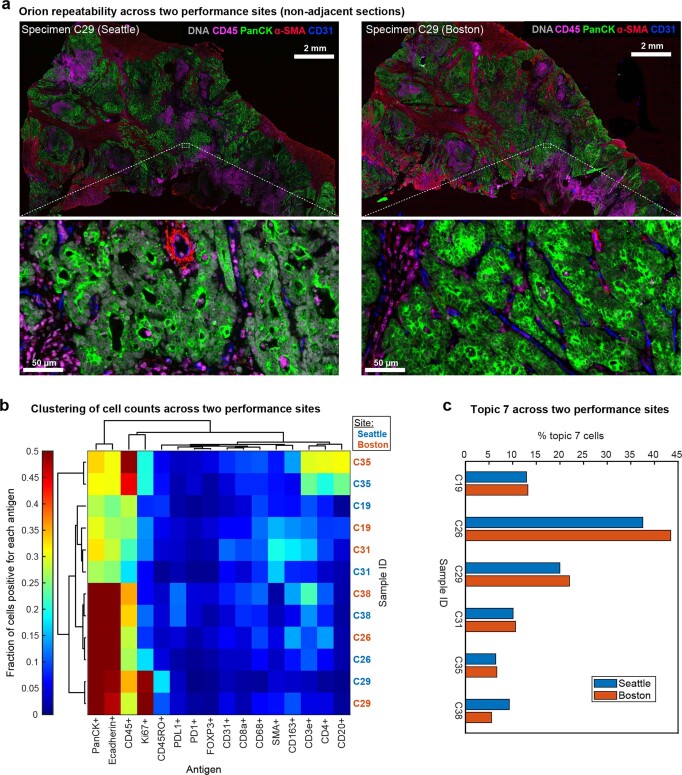
Evaluation of Orion data collected at two performance sites. **a**, Orion images of two adjacent sections acquired in two different laboratories. Specimen on the left was imaged at RareCyte, Inc in Seattle, WA and specimen on the right at HMS in Boston, MA. DNA (Sytox), CD45, pan-cytokeratin, α-SMA, and CD31 are shown. Scalebars 2 mm and 50 µm, as indicated. Images are from one representative sample (C29). **b**, Two-way hierarchical clustering heat map for the indicated markers and samples imaged at RareCyte (C19, C26, C29, C31, C35, C38) or at HMS (C19new, C26new, C29new, C31new, C35new, C38new) with the fraction of positive cells mapped to color. **c**, Bar plots showing the percentage of Topic 7 present in the indicated samples (C19, C26, C29, C31, C35, C38) imaged at two performance sites. Source data

To test the repeatability of the method, sample processing and imaging of CRC cohort 1 (*N* = 40 specimens) was performed at RareCyte, and processing and imaging of cohort 2 (*N* = 34 specimens from different individuals) was performed at Harvard Medical School (HMS) on different instruments by different operators; six specimens from cohort 1 were imaged at both RareCyte and HMS. Corresponding pairs of images from these six specimens looked very similar, and when cell count data from all 12 images were subjected to unsupervised clustering, batch effects were not observed (Extended Data Fig. 4a–c). Thus, the Orion method reproducibly generates results that are qualitatively similar to those obtained using conventional IHC, and quantitative marker intensities are similar between Orion and CyCIF.

**Extended Data Fig. 5:**
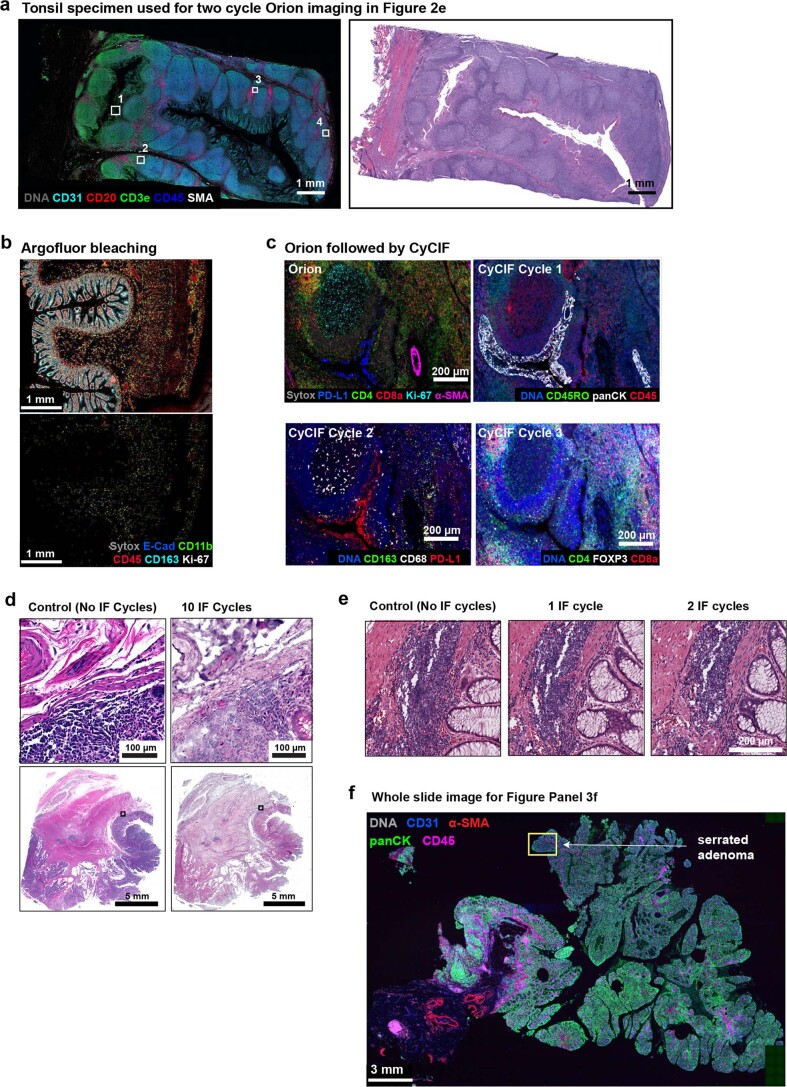
Immunofluorescence and H&E images following multiple cycles of Orion imaging. **a**, Left panel: Orion image of FFPE tonsil showing DNA (Sytox), CD31, CD20, CD3e, CD45 and α-SMA). Scalebars 1 mm. ROIs 1 to 4 displayed in Fig. 2e are noted. Scalebar 50 µm. Right panel: H&E image after two cycle Orion imaging (that is, after imaging of one panel, inactivation, and imaging of a second panel). Scalebars 1 mm. **b**, Top panel: Orion image of normal colon showing E-cadherin, CD11b, CD45, CD163, Ki-67, and DNA (Sytox) signal. Lower panel: same area of normal colon following inactivation of Orion fluorophores (see Methods). **c**, Same-slide Orion and CyCIF experiment. The tonsil samples were first processed with 16 Orion antibodies; PD-L1, CD4, CD8a, Ki-67, and α-SMA are shown. After imaging, fluorophores were inactivated by bleaching using the standard CyCIF protocol, then three-cycles of four-channel CyCIF staining and imaging were performed using the indicated antibodies. **d**, Images of H&E**-**stained sections of colorectal cancer without prior IF staining (right) and following 10 cycles of IF (left) using the standard CyCIF approach. Area shown in insets is indicated in the low magnification images. Scalebars 5 mm and 100 µm. **e**, Images of H&E**-**stained sections of colorectal cancer performed before IF imaging (0 cycles), after one cycle of IF imaging (1 IF cycle), and after two cycles of IF imaging (2 IF cycles). Scalebars 200 µm. **f**, Orion IF image from colorectal cancer resection, showing an area of serrated adenoma with low pan-cytokeratin expression (markers as indicated). Higher magnification inset as indicated by the box is shown in Fig. 3f. Scalebar 3 mm. Images are from one specimen (C26).

There are situations in which data from 16–20 fluorescent channels are likely to be insufficient for identifying cell types of interest. We therefore asked whether multiple rounds of Orion data collection could be performed on the same cells using a cyclic approach^9,14^. We stained tonsil tissue with 16 ArgoFluor-conjugated antibodies and collected IF, autofluorescence and nuclear (Hoechst-stained) images. Slides were then subjected to oxidation with hydrogen peroxide (bleaching), stained with 13 additional antibodies (a number based on reagent availability), imaged by IF and subjected to H&E staining and brightfield imaging. We found that crisp, high-SNR second-round images could be obtained using a cyclic approach, yielding a 32-plex Orion image (if same-cell H&E is included; Fig. 2e and Extended Data Fig. 5a). We confirmed that the intercycle bleaching step reduced ArgoFluor intensity by >95% and that cross-talk from one cycle to the next was therefore low (Extended Data Fig. 5b). We also established that it was possible to perform multiple rounds of CyCIF after one round of Orion (Extended Data Fig. 5c); multicycle CyCIF is slower than second-cycle Orion but potentially more flexible. Moreover, although many cycles of IF staining and bleaching reduced H&E image quality, our pathology team judged H&E images collected after two IF and photobleaching steps to be indistinguishable from controls and therefore diagnostic grade (Extended Data Fig. 5d,e). We conclude that two-cycle Orion imaging retains IF and H&E image quality, opening the door to efficient 32- to 36-plex multimodal imaging. Exploratory studies suggest room for further development of cyclic and high-plex Orion imaging.

### Integrated analysis of IF and H&E images

**Fig. 3:**
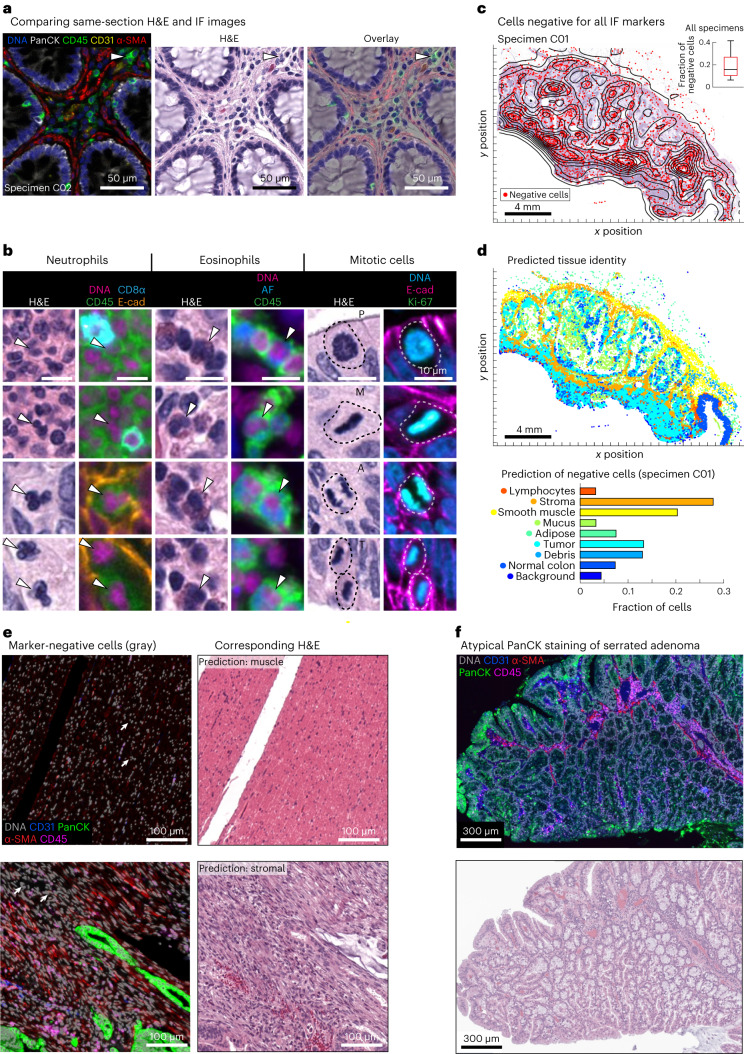
Combined H&E and Orion to identify cell and tissue types. **a**, Representative images of Orion IF and same-section H&E. All images are from one representative colorectal specimen (C02). **b**, Cell types not specifically identified by markers in the Orion panel but readily recognized in H&E images, including neutrophils, eosinophils and cells undergoing mitoses (selected cells of each type are denoted by arrowheads and dashed lines). Images are from three different representative colorectal specimens/sections (columns 1 and 2 are from C27/C04, columns 3 and 4 are from C04, and columns 5 and 6 are from C03); E-cad, E-cadherin; AF, autofluorescence; P, prophase; M, metaphase; A, anaphase; T, telophase. **c**, Spatial maps of the positions of cells (~15% of total cells) that were not detected by the Orion IF panel in a CRC specimen overlaid onto the corresponding H&E image (specimen C01); dots denote cells with identifiable nuclei but not subtyped using the antibody panel. Box and whisker plots show unidentifiable cells in cohort 1 (*N* = 40 specimens, C01–C40), the midline indicates the median, box limits indicate quartile 1 (25th percentile)/quartile 3 (75th percentile), and whiskers indicate 1.5× interquartile range (IQR). **d**, Top, spatial map of nine tissue classes determined from the H&E image using a CNN model for various cell types as indicated^36^. Bottom, percentage of the total number of ‘unidentifiable’ (negative) cells assigned to a specific tissue class by the CNN applied to the H&E image. Data were derived from *N* = 1 representative specimen (C01). **e**, Example same-section Orion IF and H&E images from areas enriched for ‘non-detected’ cells; examples include areas predicted to be rich in stroma and smooth muscle. **f**, Orion IF and H&E images showing an area of serrated adenoma with low pan-cytokeratin expression (markers are as indicated). Whole-slide image indicating the location of this region is shown in Extended Data Fig. 5f. Images are from one colorectal specimen (C26). Source data

When same-cell H&E and IF data were compared, we found that molecular labels obtained from IF enabled more complete enumeration of lymphocytes than inspection of H&E images; for example, CD4^+^ and CD8^+^ T cell and B cell lineages look similar by H&E but were distinguishable by IF (Fig. 3a). We also identified many cell types and cell states that were more readily defined in H&E images based on morphologic features than by IF staining; this included eosinophils and neutrophils with distinctive H&E morphology but no lineage markers in our Orion panels as well as the prophase, metaphase, anaphase and telophase stages of mitosis (Fig. 3b). A wide variety of acellular structures, such as basement membranes, mucin pools, necrotic domains and so on, were also more readily scored in H&E than IF images. To begin to quantify the amount of complementary information in H&E and IF images, we computed the fraction of all cells (as identified by nuclear segmentation) in the 40-specimen CRC cohort 1 that could not be assigned a clear identity using IF images; we found that this varied from 6.5 to 42% of total nuclei (median of 16%; Fig. 3c). We have previously observed a similar fraction of ‘unidentifiable’ cells following 40- to 60-plex CyCIF imaging^15^ and surmised that these cells were either negative for all antibody markers or difficult to segment^35^.

To identify cells missing labels in Orion IF data, we used a previously published ML model trained on H&E images^36^ (see Methods for details of this model and its performance). We found that >50% were predicted to be smooth muscle, stromal fibroblasts or adipocytes (Fig. 3d); these predictions were confirmed by visual inspection of the H&E images (Fig. 3e). We also examined specimens (for example, from participant 26; Fig. 3f and Extended Data Fig. 5f) in which a subset of epithelium was difficult to identify by IF because it stained weakly with pan-cytokeratin, E-cadherin and immune markers. Inspection of H&E images showed that weakly stained cells corresponded to a serrated adenoma that was distinct from a nearby domain of invasive low-grade adenocarcinoma (in which tumor cells stained strongly for pan-cytokeratin and E-cadherin). Differential staining of cytokeratin isoforms in serrated adenoma and adenocarcinoma has been described previously^37^, and we speculate that, in specimen C26, it reflects clonal heterogeneity. Low staining intensity in the serrated adenoma interferes with IF-based cell-type calling when strongly stained adenocarcinoma is also present. From these findings, we conclude that the availability of H&E and IF images of the same set of cells substantially increases the fraction of cell types and states that can be identified compared to either type of data alone. This is particularly true of cell types for which specific molecular markers do not exist (for example, stromal fibroblasts) or are not included in the panel (for example, neutrophils) and markers that are lost due to tumor subclonality (for example, specific cytokeratin isoforms). Cells that are highly elongated or have multiple nuclei and are difficult to segment (for example, muscle cells) are also commonly lost to computational analysis of IF data but highly distinctive in H&E images.

### Identifying tumor features predictive of progression

The classification of cancers for diagnostic purposes using American Joint Committee on Cancer and the Union for International Cancer Control (UICC) TNM classification criteria is based primarily on tumor-intrinsic characteristics (tumor, lymph node and metastases, the TNM staging system)^38^. However, the extent and type of immune infiltration also plays a major role in therapeutic response and survival^39^. In CRC, this has given rise to a clinical test, the Immunoscore^40^, that quantifies features of the intratumoral and tumor-proximal immune response to predict CRC progression as measured by progression-free survival (PFS) or overall survival (OS). The Immunoscore has been validated in multicenter cohort studies and predicts time to recurrence in stage III cancers in a phase III clinical trial^41^. The Immunoscore uses IHC to evaluate the number of CD3^+^ and CD8^+^ T cells at the tumor center (CT) and the invasive margin (IM; for Immunoscore, this is defined as a region encompassing 360 μm on either side of the invasive boundary; in our work, we set this to ±100 μm from the boundary)^42^. The HR (the difference in the rate of progression) between individuals with tumors containing few immune cells in both the CT and the IM (Immunoscore = 0) and individuals with tumors containing many cells in both compartments (Immunoscore = 4) has been reported to be 0.20 (95% confidence interval (95% CI) of 0.10–0.38; *P* < 10^−4^) in a Cox regression model, with increasing score correlating with longer survival^43^. This is a clinically meaningful difference that can be used to inform key treatment decisions, for example, whether or not to deliver adjuvant therapy (chemotherapy after surgery)^44^. Because chemotherapy is associated with substantial adverse effects, requires infusion or injection in a healthcare setting and is expensive, it is desirable that individuals who are unlikely to experience disease recurrence be spared the burden of adjuvant therapy.

**Fig. 4:**
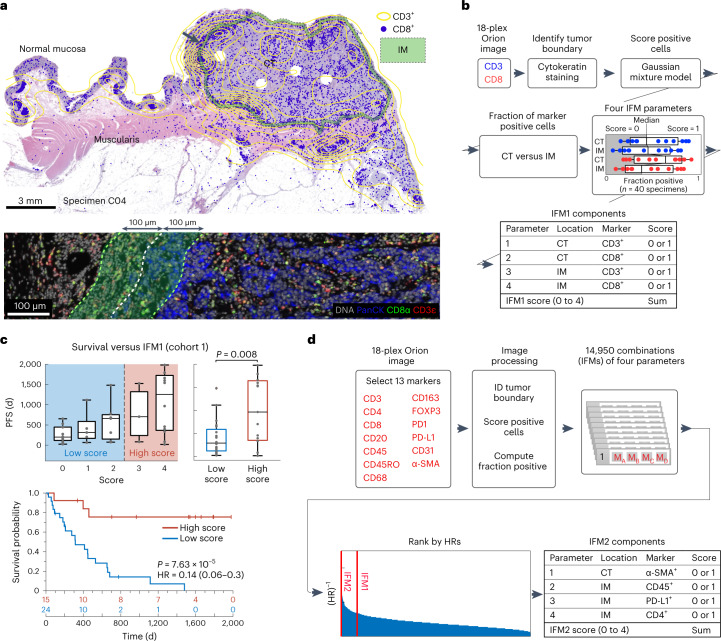
Recapitulating the Immunoscore tissue immune test using Orion images. **a**, Map of CT and IM compartments overlaid on an H&E image with the density of CD3^+^ cells shown as a contour map and the positions of CD8^+^ T cells shown as dots. The arrow indicates the zoom-in image shown below. Bottom, selected channels from a portion of the Orion image spanning the invasive boundary (denoted by shaded overlay). Images were from one representative specimen/section (C04). **b**, Flow chart for the calculation of IFM1 that recapitulates key features of the Immunoscore test. **c**, Top, box-and-whisker plots for PFS for 40 individuals with CRC based on actual IFM1 scores where the midline indicates the median, box limits indicate quartile 1 (25th percentile)/quartile 3 (75th percentile), whiskers indicate 1.5× IQR, and dots indicate outliers (>1.5× IQR). Scores are stratified into two classes as follows: low, score of ≤2; high, score of 3 or 4 (pairwise two-tailed *t*-test *P* = 0.002). Bottom, Kaplan–Meier plots computed using IFM1 binary classes (HR, 95% CI and log-rank *P* value). **d**, Flow chart for calculation of additional models that use the underlying logic of Immunoscore but considering 13 markers. The image processing steps are the same as in **a**. The rank positions of IFM1 and IFM2 are shown relative to all other 14,950 combinations of parameters that were considered. Source data

**Fig. 5:**
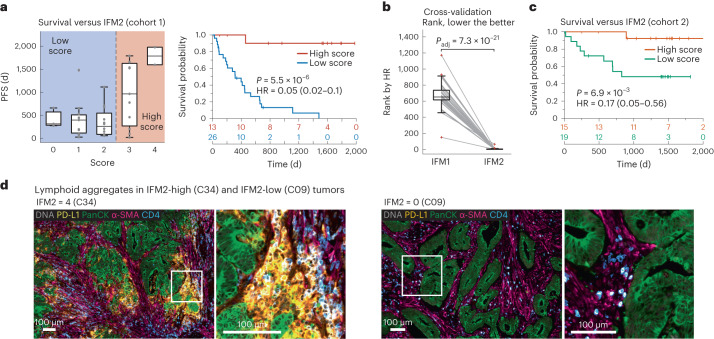
Extending the Immunoscore test with additional immune features. **a**, Left, box and whisker plots for PFS for 40 individuals with CRC based on IFM2 scores, where the midline indicates the median, box limits indicate quartile 1 (25th percentile)/quartile 3 (75th percentile), whiskers indicate 1.5× IQR, and dots indicate outliers (>1.5× IQR). Right, Kaplan–Meier plots for cohort 1 (*N* = 40 participants/specimens) computed using IFM2 binary classes (HR, 95% CI and log-rank *P* value). Scores are stratified into two classes as follows: low, score of ≤2; high: score of 3 or 4. **b**, Box-and-whisker plots of leave-one-out cross-validation of ranks from IFM1 and IFM2 (unadjusted *P* = 4.9 × 10^–26^ and adjusted using the Benjamini–Hochberg procedure *P*_adj_ = 7.3 × 10^–21^); bootstrapping of HRs is shown in Extended Data Fig. 6d. Detailed analysis procedures are described in the Methods, and pairwise two-tailed *t*-tests were used unless otherwise mentioned (*N* = 40 participants/specimens; midline indicates the median, box limits indicate quartile 1 (25th percentile)/quartile 3 (75th percentile), whiskers indicate 1.5× IQR, and dots indicate outliers (>1.5× IQR). **c**, Kaplan–Meier plot for cohort 2 computed using IFM2 binary classes stratified into two classes as follows: low, score of ≤2; high, score of 3 or 4 (HR, 95% CI and log-rank *P* value; *N* = 33 participants/specimens). **d**, Representative Orion IF images of cases with high IFM2 (score = 4) and low IFM2 (score = 0). IF images show DNA, pan-cytokeratin, α-SMA, CD45 and PD-L1. Images are from two specimens (C34 and C09), as labeled. Source data

**Extended Data Fig. 6:**
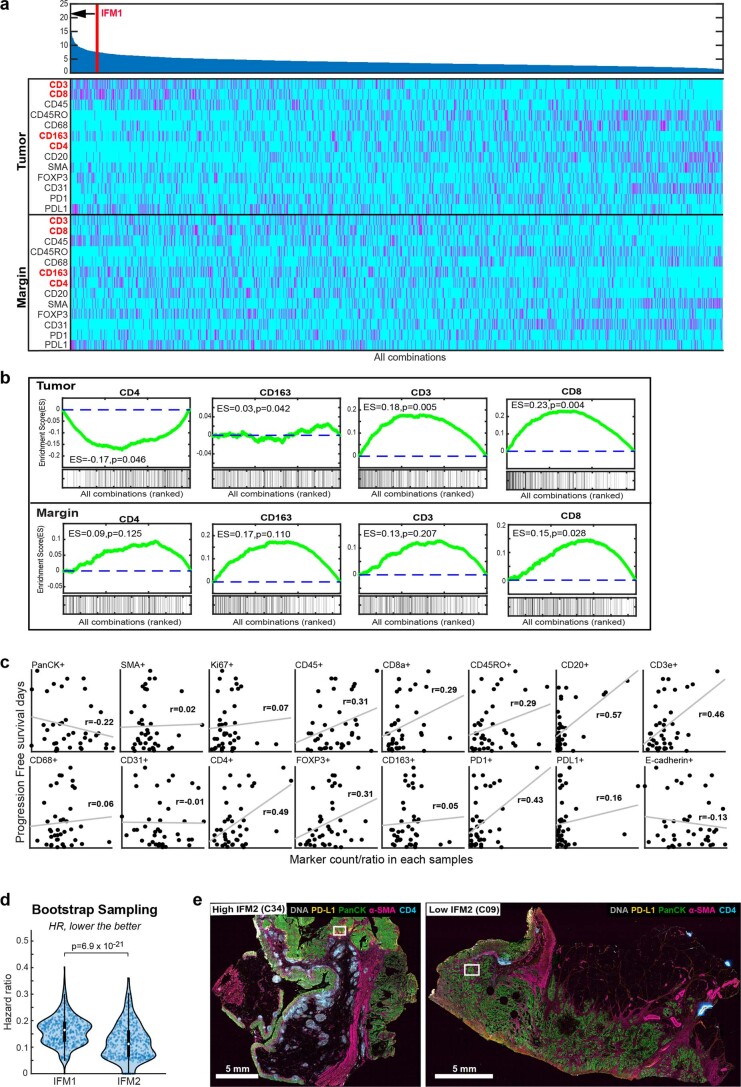
Assessment of individual markers in Image Feature Models of patient prognosis derived from Orion immunofluorescence images. **a**, Upper: Ranking of 1/hazard ratio (HR) for each Image Feature Model (IFM1 to IFM14,950) calculated by determining the positive cell frequency for one or more of 13 markers, lying within (tumor center: CT) or outside of a region 100 µm from the tumor invasive margin (IM) model (*N* = 40 patients). Ranking of IFM1 is indicated. IFM2 showed an HR = 0.08 (95% CI: 0.04–0.17, p = 1.91 × 10^−06^). Lower: Heat map showing the selected markers at the tumor or margin in each combination. 14,950 total combinations were generated as the set of 4 out of 26 parameters (13 markers in 2 regions). **b**, Enrichment plots showing enrichment scores (ES) for positive cells per indicated marker (and their location in the tumor or at the tumor margin) based on the 16-plex Orion images, indicating whether the marker/location feature is enriched in the IFMs linked to the best hazard ratios. The green lines represent the running ES for a given marker/location as the analysis proceeds down the ranked list. The value at the peak is the final ES. The Kolmogorov-Smirnov test was used to calculate the ES and p-values (*N* = 40 patients). **c**, Regression line scatter plot showing fraction of positive cells for indicated markers from the Orion 16-plex images vs. progression-free survival (PFS, days) for 40 patients with CRC. Each dot represents measurements from a single patient. r per plot is displayed. **d**, Plot bootstrapping HRs from IFM1 and IFM2 (unadjusted p = 4.62 × 10^−26^ and adjusted p = 6.9 × 10^−21^). Related to Fig. 5b. Detailed analysis is described in Methods. Pairwise two-tailed *t*-tests were used unless otherwise mentioned. Box and whisker represents *N* = 500 (random sampling), where midline = median, box limits = Q1 (25th percentile)/Q3 (75th percentile), whiskers = 1.5 inter-quartile range (IQR), and dots = outliers (>1.5IQR). **e**, Representative Orion IF images of cases with high IFM2 (IS = 4 in specimen C34) and low IFM2 (IS = 0 in specimen C09). Scalebars 5 mm. Higher magnification regions of interest shown in Fig. 5d. Images are from 2 representative patients/samples. Source data

Using Orion data, we developed software scripts to recapitulate key aspects of the Immunoscore using PFS as an outcome measure. First, we detected the tumor–stromal interface and generated masks that matched the criteria for CT and IM (±100 μm around the tumor boundary; Fig. 4a). CD3 and CD8 positivity in single cells was determined by Gaussian mixture modeling^45^, with the median positive fraction for each marker (CD3 or CD8) in each region (CT or IM) across all 40 CRC cases used as the cutoff for assigning a subscore of 0 or 1; the sum of the four subscores was used as the final score for image feature model 1 (IFM1; Fig. 4b). Parameters for computing IFM1, such as the size of the IM and the staining threshold for scoring cells positive and negative were set a priori (naively) without any parameter tuning to reduce the risk of overtraining; IFM1, nonetheless, yielded an HR similar to Immunoscore itself on cohort 1 (HR = 0.14; 95% CI of 0.06–0.30; *P* = 7.63 × 10^−5^; Fig. 4c), Next, we used the underlying logic of Immunoscore to leverage multiple Orion channels. Thirteen immune-focused markers were used to generate ~15,000 marker combinations (IFMs), each composed of four markers within the CT and IM domains (Fig. 4d). Scores for each CRC case were binarized into high and low scores based on median intensities (again without any parameter tuning). When HRs were calculated, we found that nearly 600 IFMs exceeded IFM1 in performance (Extended Data Fig. 6a–c). The top ten IFMs were insignificantly different from each other, and we chose one (IFM2) for further analysis, which exhibited an HR of 0.05 (95% CI of 0.02–0.10, *P* = 5.5 × 10^−6^; Fig. 5a) and comprised the fractions of α-smooth muscle actin (α-SMA^+^) cells in the CT and CD45^+^, PD-L1^+^ and CD4^+^ cells in the IM. Leave-one-out resampling showed that IFM2 was significantly better than IFM1 with respect to HR (adjusted *P* value (*P*_adj_) based on the Benjamini–Hochberg procedure; *P*_adj_ = 7.3 × 10^−21^; Fig. 5b and Extended Data Fig. 6d). To determine whether IFM2 is generalizable, we tested the performance of this model created using cohort 1 on specimens in cohort 2. Once again, we observed a statistically significant discrimination between progressing and non-progressing tumors (HR = 0.17; 95% CI of 0.05 to 0.56; *P* = 6.9 × 10^−3^; Fig. 5c). We conclude that multiplexed immunoprofiling data extracted from Orion images of CRC resections can be used to generate performant prognostic biomarkers.

Inspection of images from IFM2 high tumors exhibiting slow progression (for example, participant C34) revealed high numbers of PD-L1^+^ cells (Fig. 5d and Extended Data Fig. 6e) adjacent to pan-cytokeratin^+^ tumor cells; based on overlap of PD-L1, CD68 and CD45 staining, we conclude that PD-L1^+^ cells are likely myeloid in origin, as described previously^15^. In C34, α-SMA-stained tumor-proximate stromal cells (most likely fibroblasts) were also infiltrated with CD4^+^ T cells. By contrast, in a participant with rapid progression (that is, participant C09), PD-L1 levels were below the level of detection, and CD4^+^ cells were less abundant in the stroma. By H&E, IFM2-high tumors exhibited extensive lymphohistiocytic chronic inflammation, including large lymphoid aggregates and tertiary lymphoid structures (TLS) at the tumor IM^46^, whereas IFM2-low tumors had relatively few lymphoid aggregates and no TLS (Fig. 5d and Extended Data Fig. 6e). Although IFM2-low tumors were also more invasive than IFM2-high tumors, IFM score was independent of histologic subtype (for example, conventional versus mucinous morphology) and did not correlate with histologic grade (low- versus high-grade carcinoma). Thus, IFM2 is likely to capture activity of the immune microenvironment around the tumor IM as well as changes in tumor-associated fibroblasts. However, deeper phenotyping of more specimens will be required to identify precisely which molecular features of IFM2 are important for predicting progression.

### Identifying new progression markers

**Fig. 6:**
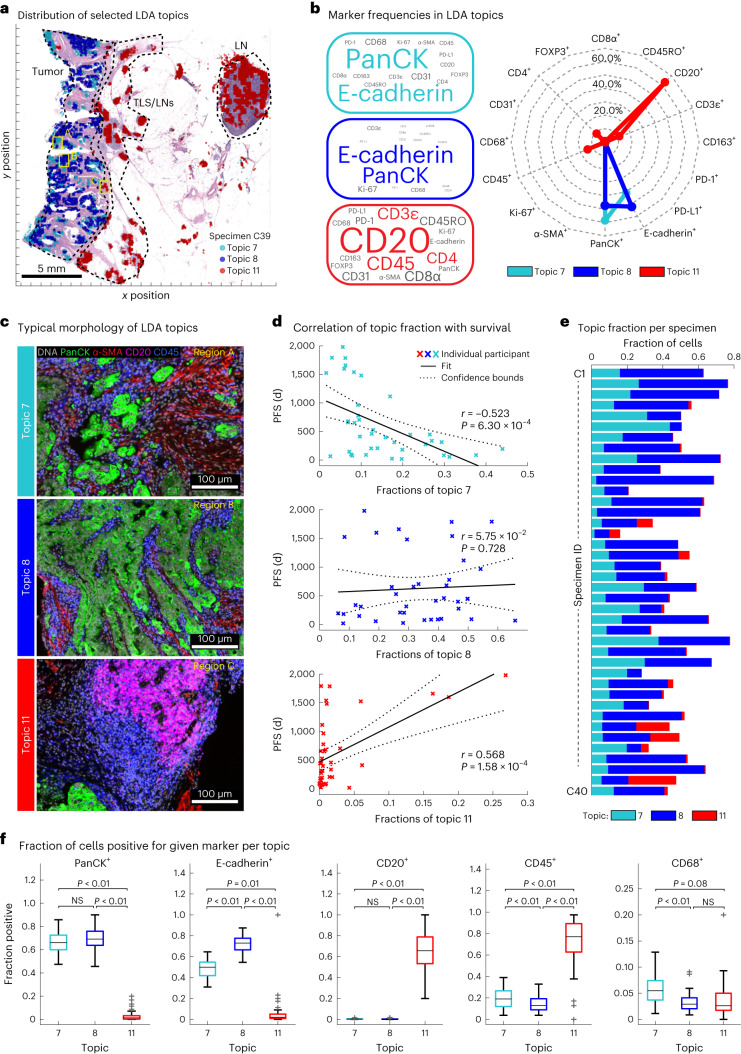
Bottom-up development of a tumor-intrinsic IFM. **a**, Positions of three selected topics identified using LDA. Topic locations are overlaid on an H&E image. Data were derived from one representative specimen (C39); LN, lymph node. **b**, Left, markers making up selected LDA topics as shown by size of the text proportional to the frequency of the marker but with colored text scaled by 50% for clarity. Right, radar plot indicating the fraction of cells positive for each marker in topics 7, 8 and 11 (data for all others topics shown in Extended Data Fig. 7). **c**, IF images showing expression of pan-cytokeratin, α-SMA, CD20 and CD45 for the indicated LDA topics. The position of each image frame is denoted by the labeled boxes in **a**. Images are from one representative specimen (C39). **d**, Pearson correlation plots of PFS and fraction of topics 7, 8 and 11 in 40 individuals with CRC. Topic 11 corresponded to TLS, whose presence is known to correlate with good outcome^68^. Pearson correlation was used, and unadjusted *P* values are provided. **e**, Fraction of topics 7, 8 and 11 in CRC specimens C01–C40. **f**, Box and whisker plots showing fractions of topic 7-, 8- and 11-positive cells for indicated markers; the midline indicates the median, box limits indicate quartile 1 (25th percentile)/quartile 3 (75th percentile), whiskers indicate 1.5× IQR, and dots indicate outliers (>1.5× IQR). Two-tailed pairwise *t*-test *P* values are indicated (*N* = 40 participants/specimens). The *P* values are listed below; pan-cytokeratin^+^: 2.83 × 10^−44^ (7 versus 11), 0.12 (7 versus 8), 4.48 × 10^−42^ (8 versus 11); E-cadherin^+^: 2.4 × 10^−21^ (7 versus 11), 8.26 × 10^−21^ (7 versus 8), 1.22 × 10^−30^ (8 versus 11); CD20^+^: 1.99 × 10^−23^ (7 versus 11), 0.63 (7 versus 8), 1.94 × 10^−23^ (8 versus 11); CD45^+^: 3.99 × 10^−18^ (7 versus 11), 6.7 × 10^−3^ (7 versus 8), 1.6 × 10^−19^ (8 versus 11); CD68^+^: 0.084 (7 versus 11), 2.88 × 10^−5^ (7 versus 8), 0.28 (8 versus 11); NS, not significant. Source data

**Extended Data Fig. 7:**
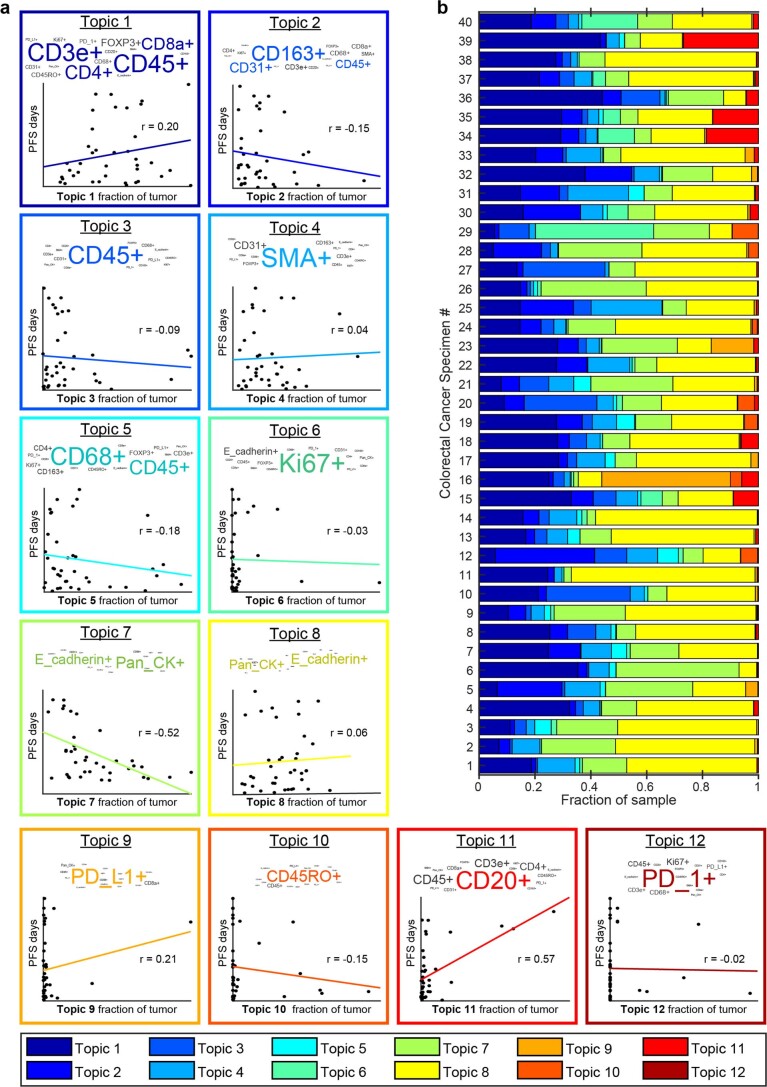
Cellular neighborhoods in colorectal cancer resections. **a**, Latent Dirichlet Allocation (LDA) probabilistic modeling was used to analyze Orion immunofluorescence data from 40 colorectal cancer specimens to reduce cell populations into neighborhoods (‘topics’) defined by patterns of single-cell marker expression. The analysis identified 12 topics that recurred across the dataset. Within each box is the LDA plot for the indicated topic (top) and a regression line scatter plot indicating the fraction of each tumor composed of the indicated LDA topic and the relationship to progression-free survival (PFS, days). Each dot represents measurements from a single patient. r value for each plot is displayed. **b**, Bar plot depicting the proportional distribution of the LDA Topics in the 40 colorectal cancer specimens. Source data

As an unbiased means of identifying new progression models, we used spatial latent Dirichlet allocation (Spatial-LDA)^47^. Spatial-LDA can reduce complex assemblies of intermixed entities into distinct component communities (‘topics’) while accounting for uncertainty and missing data; it has performed well on other multiplexed tissue imaging datasets^48,49^. We separated CRC specimens in cohort 1 into tumor and adjacent normal tissue using H&E data and an ML/AI model^36^ and performed Spatial-LDA at the level of individual IF markers on cells in the tumor region (Fig. 6a). This yielded 12 spatial features (topics) that recurred across the dataset (the number of topics was optimized by calculating the perplexity; Extended Data Fig. 7a and Methods). Visual inspection of images by a pathologist confirmed that marker probabilities matched those computed for different topics and that the frequency distribution of each topic varied, sometimes substantially, among CRC samples (Fig. 6b and Extended Data Fig. 7b). The strongest correlations between topics and PFS for cohort 1 were found to be −0.52 (*P* < 0.001) for topic 7, comprising pan-cytokeratin and E-cadherin positivity (with little contribution from immune cells), and +0.57 (*P* < 0.001) for topic 11, comprising CD20 positivity with minor contributions from CD3, CD4 and CD45 (Fig. 6b–f and Extended Data Fig. 7a). By contrast, topics involving the proliferation marker Ki-67 (topic 6), PD-L1 positivity (topic 9) or immune cell markers (CD45 or CD45RO; topics 3 and 10) exhibited little or no correlation with PFS (Extended Data Fig. 7a).

**Fig. 7:**
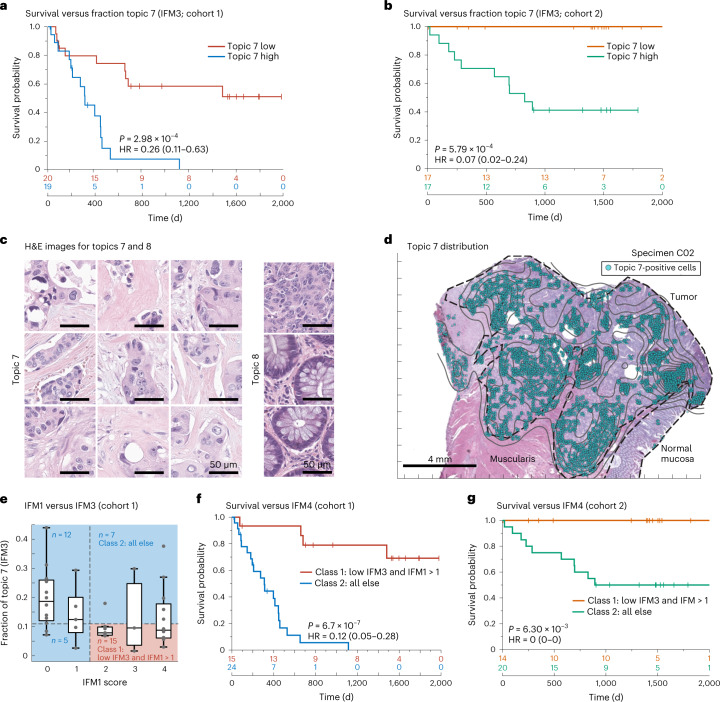
LDA topic 7 corresponds to aggressive tumor regions and is correlated with poor outcomes. **a**,**b**, Kaplan–Meier plots of PFS based on the fraction of topic 7 present in the tumor domain and stratified as ‘high’ when above the median (50th percentile) and ‘low’ when below the median of all cases (HR, 95% CI and log-rank *P* value) for 40 individuals with CRC in cohort 1 (**a**) and 34 individuals with CRC in cohort 2 (**b**). **c**, Representative H&E images of topic 7 (left) and topic 8 (right) extracted from all specimens using a CNN (GoogLeNet) trained on LDA data. Images were derived from 10 participants/specimens (C01–C10). **d**, Spatial map of LDA topic 7 and H&E image for one representative specimen (C02). **e**, Plot of fraction of topic 7 (IFM3) versus IFM1 score for 40 individuals with CRC. The midline indicates the median, box limits indicate quartile 1 (25th percentile)/quartile 3 (75th percentile), whiskers indicate 1.5× IQR, and dots indicate outliers (>1.5× IQR). **f**,**g**, Kaplan–Meier plots stratified using IFM4, which was binarized as follows: class 1, IFM1 high and topic 7 (IFM3) low group; class 2, all other participants (that is, either low IFM1 and/or high topic 7 (IFM3); HR, 95% CI and log-rank *P* value) for cohort 1(40 individuals with CRC; **g**) and cohort 2 (34 individuals with CRC; **h**). Source data

**Extended Data Fig. 8:**
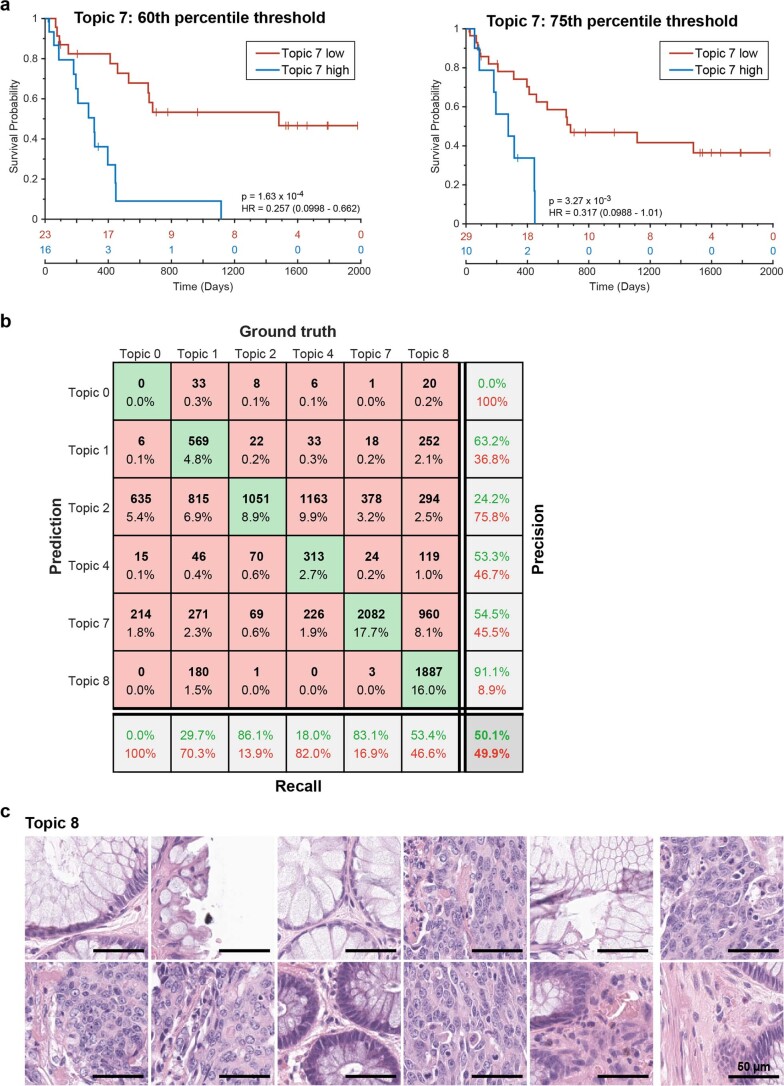
Evaluation of the performance of a Convolution Neural Network used to identify cellular neighborhood Topic 7 from H&E images of colorectal cancer. **a**, Kaplan Meier plots of PFS for 40 CRC patients based on the fraction of Topic 7 present in the tumor domain and stratified using a threshold (‘cutoff’) of 60^th^ percentile (left) and 75^th^ percentile (right) (HR, hazards ratio; 95% confidence interval; logrank p-value). **b**, Confusion matrix table showing performance of GoogLeNet convolutional neural network (CNN) trained using H&E data from Latent Dirichlet Allocation (LDA) Topic 7 and its performance in identifying Topic 7 cells from H&E data. Topic 0 contains the rest of the topics (3, 5, 6, 9, 10, 11, 12). Target class (ground truth) was assigned from LDA analysis of Orion images and Output class (predicted) was assigned by the GoogLeNet CNN. **c**, Gallery of representative H&E images of true positives for topic 8 from 10 patients/specimens (C01-C10); Scalebars 50 µm. Source data

Given the correlation of topic 7 with PFS, we constructed a Kaplan–Meier curve for tumors having a proportion of topic 7 below the 50th percentile versus those above this threshold (including all cells in the specimen; note that the value of the threshold was not critical over the range of 50–75% (Fig. 7a and Extended Data Fig. 8a). Imposing a 50th percentile threshold on topic 7 yielded model IFM3, which, on cohort 1, resulted in an HR of 0.26 (Fig. 7a; 95% CI of 0.11–0.63; *P* = 2.98 × 10^−4^). When we tested IFM3 on cohort 2, we observed even better performance (HR = 0.07; 95% CI of 0.02–0.24; *P* = 5.6 × 10^−4^; Fig. 7b), suggesting that the model had not been overtrained. We conclude that Spatial-LDA had discovered (via unsupervised analysis of high-plex IF data) a tumor-intrinsic feature distinct from immune infiltration that was significantly associated with poor survival.

**Extended Data Fig. 9:**
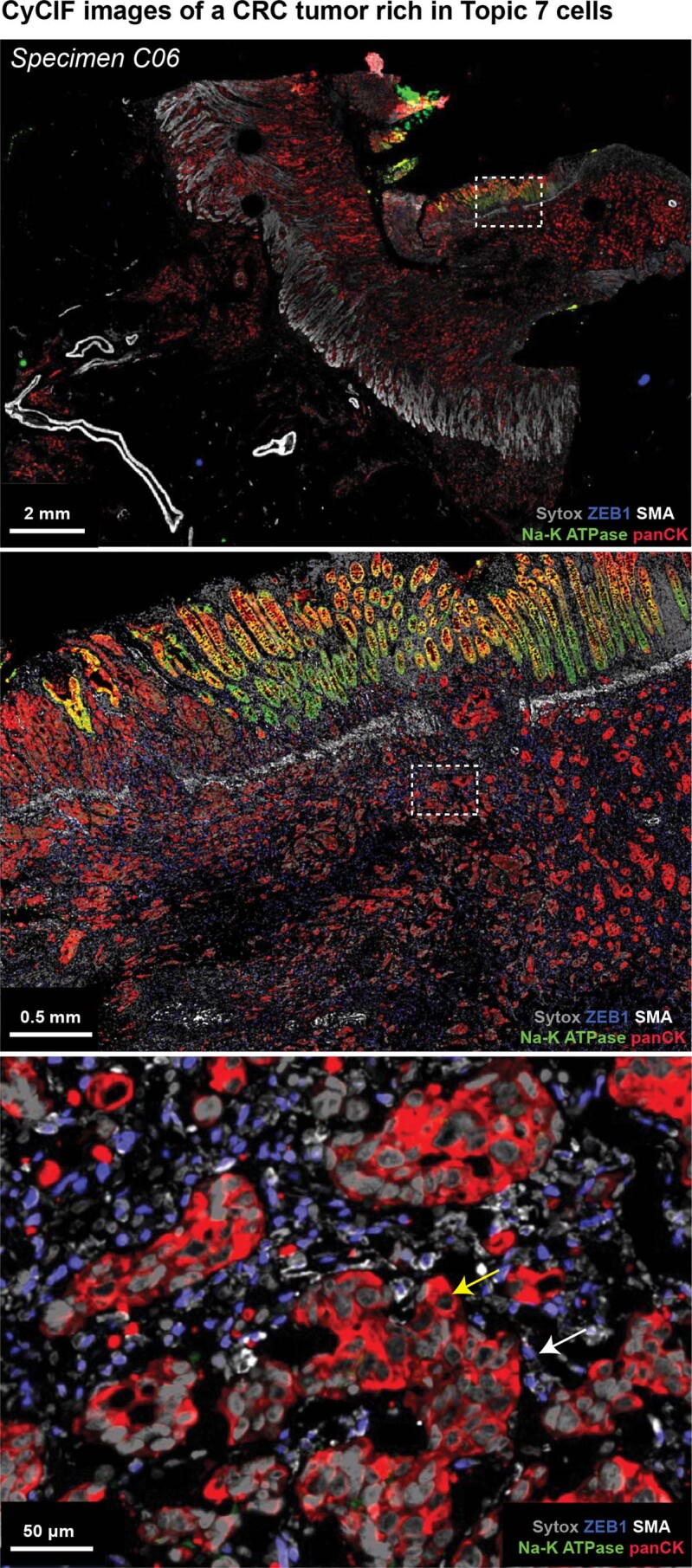
CyCIF imaging of Topic 7 tumor cells. CyCIF imaging of regions of specimen C06 which had a high fraction of Topic 7 cells. The CyCIF image is from a non-adjacent section to that used for Orion data section. Images show DNA (Sytox), ZEB1, α-SMA, NA-K ATPase, pan-cytokeratin. Location of insets are indicated. Scalebars, 2 mm, 0.5 mm, 50 µm, as indicated. The mesenchymal differentiation/EMT-marker ZEB1 (nuclear blue signal) is present in stromal cells (white arrow) but absent in the tumor cells (marked by pan-cytokeratin, red; yellow arrow). Images here are from one sample (C06).

Poor interpretability is a common limitation of models generated using ML methods such as Spatial-LDA. In the case of topic 7, the primary molecular features were pan-cytokeratin and E-cadherin positivity, but topic 8 was similar in composition while exhibiting no correlation with PFS (*r* = 0.01; Fig. 6c,f and Extended Data Fig. 7a). To identify the tumor histomorphology corresponding to these topics, we transferred labels from IF to the same-section H&E images, trained a convolutional neural network (CNN) on the H&E data and inspected the highest-scoring tumor regions (Extended Data Fig. 8b). Topic 7 was readily identifiable as poorly differentiated/high-grade tumor with stromal invasion (Fig. 7c,d). By contrast, topic 8 consisted predominantly of nearly normal intestinal mucosa with some areas of well-differentiated tumor (Fig. 7c and Extended Data Fig. 8c). When we inspected Orion and CyCIF images of specimens with a high proportion of topic 7 (for example, participant C06; Extended Data Fig. 9), we found that the E-cadherin-to-pan-cytokeratin ratios were low relative to normal mucosa or topic 8 (expression of Na^+^/K^+^-ATPase, another protein found on the plasma membranes of colonic epithelial cells, was also low). These are features of cells undergoing an epithelial–mesenchymal transition (EMT), which is associated in CRC with progression and metastasis^50^. However, follow-on CyCIF imaging showed that some features of EMT, such as low proliferation and increased expression of EMT-associated transcriptional regulators (for example, ZEB1), were not generally observed in topic 7-positive cells. For example, the proliferation index in tumor cells was high (40–50% Ki-67 and PCNA positivity), and staining for ZEB1 was low (although ZEB1 was easily detected in nearby stromal cells with mesenchymal differentiation; Extended Data Fig. 9). Thus, although the molecular and morphological features of topic 7 were consistent with each other, H&E morphology was more readily interpretable than long-established features of CRC progression. Interpretability increases confidence in a potential biomarker and substantially improves its chances of clinical translation^51^.

Only about one-third of participants in cohort 1 scored high for IFM1 and low for IFM3 (the combination correlated with the longest PFS; Fig. 7e), arguing for use of a composite model (IFM4; HR = 0.12; 95% CI of 0.05–0.28; *P* = 6.7 × 10^−7^; Fig. 7f). Statistically significant results were also obtained from cohort 2 using a model trained on cohort 1 (Fig. 7g). This demonstrates that immunological and tumor-intrinsic features of cancers can be combined to generate prognostic models with high predictive value. Although no parameter tuning (for example, setting thresholds for positivity) was involved in the generation of IFMs 1–4, experience with Immunoscore shows that parameter tuning using larger cohorts of individuals can further boost performance.

## Discussion

In this paper, we describe an approach to multimodal tissue imaging that combines one cycle (single-shot), high-plex subcellular-resolution IF with imaging of endogenous fluorescence and subsequent H&E imaging of the same cells. We show that such multimodal tissue imaging is reproducible across performance sites and has substantial benefits for human observers and ML models. Most obviously, it facilitates the use of historical knowledge about tissue microanatomy (derived from H&E images) for the interpretation of molecular data derived from multiplexed molecular imaging. We find that human experts and ML algorithms can exploit H&E images to classify cell types and states that are not readily identifiable in multiplexed data given inevitable limitations in antibody variety. H&E and autofluorescence imaging are also effective at characterizing acellular structures that organize tissues at mesoscales (for example, the elastic lamina of the vessel wall). Moreover, by overlaying molecular data on H&E images, we show that it is possible to discriminate cell types that have similar morphologies but different functions. The ability of molecular data to label cell types in H&E images is expected to be advantageous in supervised learning for ML/AI modeling^6^ as is the use of H&E data to unpack ‘black box’ ML models trained on molecular data. The interpretability of AI models is thought to be important in medicine as a means of promoting uptake, increasing generalizability across cohorts and ensuring compliance with ethical standards^52^.

The Orion instrument described here supports up to 18-plex simultaneous data acquisition (including DNA and one or more autofluorescence channels), but it is likely that several additional channels can be added as fluorophores are more optimally matched to available lasers and optical elements. Our data demonstrate 18-plex Orion imaging of 30 types of cancer, diseased tissues and normal tissues available as TMA cores or whole-slide specimens, demonstrating that the Orion method is widely applicable. Of course, the antibody panel used for CRC will not be optimal for all tissues, but substitution of a few antibodies is expected to yield panels usable with many cancers of epithelial origin. The only practical limitation to assembling additional Orion panels using commercial antibodies developed for IHC and IF imaging of tissues is the time needed to prepare ArgoFluor-labeled antibodies and test panel performance and stability.

We show that it is possible to perform cyclic data acquisition using the Orion approach as well as Orion followed by CyCIF, thereby increasing the number of molecular channels dramatically. Cyclic Orion is particularly well suited to discovery research in which 20- to 40-plex imaging is increasingly common^53^. However, H&E staining must be performed after all IF is complete, and we find that H&E image quality goes down as IF data acquisition extends beyond two to four cycles. For diagnostic applications, our data suggest that image-based prognostic tests may require only a subset of the channels available to Orion (speculatively 8–14 channels) with attendant reductions in test complexity and cost.

It is not surprising that multiplexed IF data add information to H&E images. More surprising are the many cell types and structures that are difficult to identify in multiplexed images and are readily identified in H&E images by histopathologists or ML algorithms. These include acellular structures, cell types for which good markers are not readily available, highly elongated and multinucleated cells that are difficult to segment with existing algorithms (for example, muscle cells) and, most remarkably, tumor cells themselves. Many tumor types lack a definitive cell-type marker, and even when such markers are available, some cells in a tumor are observed to express these markers poorly or not at all, likely due to subclonal heterogeneity^54^. By contrast, pathologists are skilled at identifying dysplastic and transformed cells in H&E images based on morphology, and this is potentially more reliable than IF imaging using molecular markers for the identification of some types of tumor cells. Conversely, many immune cell types cannot be reliably differentiated using H&E images, and their presence can also be difficult to discern when cells are crowded; the use of IF lineage markers provides critical new information in these cases.

There are multiple ways to exploit the complementary strengths of H&E and IF imaging using ML approaches. ML models trained on H&E data can increase the number of identifiable cells in multimodal images relative to multiplexed IF data alone. Conversely, IF images can be used to automatically label structures in H&E images (for example, immune cell types) to assist in supervised learning on these images. This is a notable development because the labor associated with labeling of images (currently by human experts) is a barrier to the development of better ML models. Finally, multimodal data can provide molecular insight into the features of tissue images to which ML models ‘attend’^55^. We anticipate many opportunities for joint learning from H&E and IF data using adversarial, reinforcement and other types of ML/AI modeling for research purposes, development of biomarkers and analysis of clinical H&E data at scale^5^.

A surprising number of pathology workflows involve staining serial sections of a specimen with one IHC biomarker each; the Orion approach can simplify such workflows to single-section imaging. For example, Immunoscore is a pathology-driven clinical test that uses H&E and multiple IHC sections to determine the distribution of specific immune cell types at the tumor margin and predict outcome (time to recurrence) for individuals with CRC. In this paper, we reproduced the logic of Immunoscore with Orion data plus automated scripts and show that it is possible to improve on it using additional immune markers (as measured by HRs computed from PFS data; see limitations section below)^56^. In a distinct but complementary approach, we show that IF data and spatial modeling (LDA) can be used to identify cell neighborhoods significantly associated with CRC progression. The top-performing feature in this case is tumor cell intrinsic and based on the distributions of cytokeratin and E-cadherin, two epithelial cell markers. Why exactly this feature is prognostic is unclear from IF data alone; other features involving similar markers are not predictive. However, inspection of corresponding H&E data (and training of an ML model) showed that LDA had identified local tumor morphologies typical of poorly differentiated/high-grade tumors with stromal invasion, increasing our confidence in the model. The immediate availability of Orion as a commercial platform and our use of open-source software and Open Microscopy Environment-compliant^57^ and Minimum Information about Tissue Imaging-compliant^58^ data standards make further development of these approaches straightforward.

Although the images in this paper represent a large dataset by the standards of high-plex whole-slide IF imaging, the number of specimens and the composition of the cohort is insufficient for IFMs to be considered validated biomarkers or clinical tests. Systematic meta-analysis has identified a range of factors that negatively impact the reliability and value of prognostic biomarkers^59^, particularly those based on new technology and multiplexed assays. In the current work, specific limitations include a relatively small cohort size, the absence of preregistration and the acquisition of specimens from a single institution. As a result, we do not fully control for all relevant covariates (for example, depth of invasion, sex, age, race, clinical stage and so on), and more progressors were included in our cohort than would be observed in an unselected population, biasing the cohort to more serious disease (~50% 2-year disease-free survival for stage III colon cancer in our cohort versus an accepted value of ~80%) (ref. ^60^). These and other limitatons are addressable with more diverse sets of tissue blocks, and we anticipate that it will be feasible to progress in a few years to validated clinical tests that can be added to CRC treatment guidelines^44^, substantially improving opportunities for personalized therapy.

## Methods

### Ethics and tissue cohort

The research described in this manuscript complies with all relevant ethical regulations and was reviewed and approved by the Institutional Review Boards (IRBs) at BWH, HMS and Dana Farber Cancer Institute (DFCI). FFPE tissue samples were used after diagnosis, and informed written consent was acquired under DFCI IRB protocol 17-000 and a discarded excess tissue protocol. Two cohorts from the same biobank were assembled, the first with 40 individuals with stage II–IV CRC and the second with 34 individuals with no consideration for the sex of the participants. All samples were collected at the time of initial diagnosis.

### Tissue preparation

Blocks of FFPE tonsil (AMSBIO, 6022CS) and lung adenocarcinoma (AMSBIO, 28004), and multi-tissue TMA (HTMA427) and colorectal adenocarcinomas from the BWH Pathology Department archives were cut at 5-µm thickness using a rotary microtome, and the sections were mounted onto Superfrost Plus microscope glass slides (Thermo Fisher, 12-550-15). The slides were dried at 37 °C overnight and baked at 59 °C for 1 h. Slides were stored at 4 °C until use.

### Fluorophores for Orion imaging

The Orion instrument is designed to work with optimized ArgoFluor dyes (RareCyte) whose emission peaks cover the spectrum from green to far red (Supplementary Table 2). Although the instrument can also be used with other commercially available dyes, ArgoFluor dyes have been strategically chosen based on resistance to photobleaching, narrow excitation and emission spectra and high quantum efficiency. To date, RareCyte has optimized 18 ArgoFluor dyes, with others in development.

### IF antibodies

Antibodies (listed in Supplementary Table 2) were obtained from vendors in carrier-free PBS and were conjugated directly to biotin for α-SMA and digoxygenin for pan-cytokeratin or to ArgoFluor dyes using amine conjugation chemistry. Labeling efficiency was determined using absorbance spectroscopy, and the conjugated antibodies were diluted in PBS-antibody stabilizer (CANDOR Bioscience, 130050) to a concentration of 200 µg ml^–1^.

### IF staining

Slides were deparaffinized and subjected to antigen retrieval for 5 min at 95 °C followed by 5 min at 107 °C using EZ-AR 2 Elegance buffer (pH 8.5; BioGenex, HK547-XAK). To reduce tissue autofluorescence, slides were placed in a transparent reservoir containing 4.5% hydrogen peroxide and 24 mM NaOH in PBS and illuminated with white light for 60 min followed by 365-nm light for 30 min at room temperature, as previously described^14^. Slides were rinsed with surfactant wash buffer (0.025% Triton X-100 in PBS), placed in a humidified stain tray and incubated in Image-iT FX signal enhancer (Thermo Fisher, I36933) for 15 min at room temperature. After rinsing with surfactant wash buffer, the slides were placed in a humidity tray and stained with the panel of fluor- and hapten-labeled primary antibodies in PBS-antibody stabilizer (CANDOR Bioscience, 130050) containing 5% mouse serum and 5% rabbit serum for 2 h at room temperature. Slides were then rinsed again with surfactant wash buffer, placed in a humidified stain tray and incubated with Hoechst 33342 (Thermo Fisher, H3570), ArgoFluor 845 mouse anti-DIG and ArgoFluor 875-conjugated streptavidin in PBS-antibody stabilizer containing 10% goat serum for 30 min at room temperature. The slides were rinsed a final time with surfactant wash buffer and PBS, coverslipped with ArgoFluor mounting medium (RareCyte) and dried overnight.

### ArgoFluor–antibody conjugate stability testing

Antibody accelerated aging studies were performed to determine ArgoFluor–antibody conjugation stability. Reagent stability was measured using the ratio of quantitative metrics obtained with the accelerated condition (21.6 °C) to those obtained with the storage condition (−20 °C). For testing on tissues, single-cell mean fluorescence intensity (MFI) data obtained by imaging FFPE tonsil tissue stained with an ArgoFluor conjugate were gated using a Gaussian mixture model to obtain the percentage of positive cells and signal:background (S:B) values (S and B refer to the MFI of cells with values above (S) and below (B) the gated threshold). To perform fluorophore stability assessments, bead MFI data were obtained by imaging immunoglobulin capture beads incubated with (S) or without (B) the ArgoFluor conjugate. For binding stability assessments, data from peripheral blood mononuclear cells stained with the ArgoFluor-conjugated antibody were assessed in a fluorescence-activated cell sorter and manually gated to obtain the percentage of positive cells and S:B values.

### The Orion method and instrumentation

In the Orion instrument, ArgoFluor-conjugated antibodies along with Hoechst dye and tissue autofluorescence were excited by seven laser lines, ranging from 405 to 730 nm (Supplementary Table 2). To separate the overlapping emission spectra, images were captured through a set of nine bandpass filters, which can achieve a tunable narrow band detection window (10–15 nm) throughout the spectrum from 425 nm to 894 nm. For a specific sample, the detection bands were chosen to optimize color separation, implemented with RareCyte’s Artemis software. Tuning of emission filters is based on the well-known fact that the spectrum of a thin-film interference filter shifts toward shorter wavelengths when the angle of incidence shifts away from 0° (orthogonal to the filter surface). The filters were motorized such that any narrow band of 10–15 nm can be achieved across the entire fluorescence spectrum. Narrow bandpass emission channels improve specificity; the resulting lower signal is overcome by using high-power excitation lasers, which yield power at the sample plane ranging from 270 to 600 mW, more than ten times greater than a typical fluorescence microscope.

### Considerations in the development of Orion antibody panels

High-plex imaging exploits the fact that the greater the number of features collected, the greater the ability to distinguish lineages and states at a single-cell level. The ability of the Orion imaging platform to discriminate among multiple antibody–fluorophore conjugates is dependent on the degree of spectral overlap among the fluorophores, the intensity and spectral profile of overlapping autofluorescence or background signals and the difference between the most intense staining of highly expressed proteins and the weakest stain of low-abundance proteins. Panel design with the Orion platform involves assigning biomarkers to channels with the appropriate sensitivity ranges while managing spectral overlap between markers that are colocalized. Orion imaging technology is compatible with 20-plex one-shot fluorescence image acquisition (19 antibodies plus Hoechst nuclear stain), and the necessary research into ArgoFluor is ongoing to achieve this on a routine basis. In the current work, we found that 17-plex antibody panels were easier to achieve at an acceptable SNR given the endogenous fluorescence of tonsil and CRC tissue. We anticipate that, with relatively few additions and substitutions, the panel we developed for CRC will work well with other common tumor types (for example, lung, breast and melanoma). In cases in which more precise immunophenotyping is desired, a second-cycle Orion panel of similar complexity is possible. The prognostic IFMs we describe in this paper could be acquired using as few as 8–12 channels. Thus, optimal Orion imaging and staining strategies in both research and clinical settings are likely to rely on the use of both preset high-plex and lower-plex lower-cost ‘mix and match’ panels.

### One-shot antibody IF imaging with the Orion instrument

Whole slides were scanned on the Orion instrument using acquisition settings optimized for the specific antibody panels. Briefly, acquisition channel parameters were defined for each biomarker plus an additional channel dedicated to tissue autofluorescence and included excitation laser, emission CWL and exposure times. The nuclear channel was scanned at low resolution to identify tissue boundaries, followed by surface mapping at ×20 to find the tissue in the *z* axis. Whole tissue was acquired at ×20 following the surface map within the specified tissue boundaries by collecting all channels for a single field of view (FOV) before proceeding to the next partially overlapping FOV. Raw image files were processed to correct for system aberrations, and signals from individual targets were then isolated using the spectral matrix obtained with control samples, followed by stitching of FOVs to generate a continuous open microscopy environment pyramid TIFF image.

### Same-section H&E staining and imaging

After Orion imaging was complete, slides were decoverslipped by immersion in 1× PBS at 37 °C until the coverslips fell away from the slide. Slides were rinsed in distilled water for 2 min and stained by a routine regressive H&E protocol using Harris hematoxylin (Leica, 3801575) and alcoholic eosin Y (Epredia, 71211). Coverslipping was performed with toluene-based mounting medium (Thermo Scientific, 4112). After drying for 24 h, slides were scanned on an Orion system in brightfield mode using the same scan area used for IF image acquisition. H&E images were also acquired using an Aperio GT450 microscope (Leica Biosystems), and the H&E images were registered to the IF images using ASHLAR^61^ and PALOM v2022.3 (https://github.com/labsyspharm/palom) software.

### Pathology annotation of H&E images

H&E images were annotated by a board-certified anatomic pathologist (S.C. and S.S.) in OMERO PathViewer software on whole-slide images according to morphologic criteria^62^ that included normal mucosa, hyperplastic mucosa, adenomatous mucosa (tubular or serrated), invasive adenocarcinoma (tumor), lymphovascular invasion, perineural invasion, secondary lymphoid structures/Peyer’s patches, TLSs, lymphoid aggregates (without identifiable germinal center formation) and lymph nodes. TLSs were morphologically defined by the presence of a lymphoid aggregate with germinal center formation and an anatomic distribution and appearance inconsistent with a secondary lymphoid structure (Peyer’s patch or lymph node).

### CyCIF imaging

Tissue-based CyCIF was performed as previously described^14^ following the methods available in protocols.io (10.17504/protocols.io.bjiukkew)^63^. Data from specimens C01–C17 were acquired as previously reported^15^, and computed cell counts were compared in this study to cell counts derived from Orion images of adjacent sections from the same specimens. A BOND RX automated slide stainer (Leica Biosystems) was used to bake FFPE slides at 60 °C for 30 min. Dewaxing was performed using Bond Dewax solution at 72 °C, and antigen retrieval was performed using BOND epitope retrieval solution 1 at 100 °C for 20 min. Slides then underwent multiple cycles of antibody incubation, imaging and fluorophore inactivation to perform the CyCIF process. All antibodies were incubated overnight at 4 °C in the dark. Slides were stained with Hoechst 33342 for 10 min at room temperature in the dark following antibody incubation in every cycle. Coverslips were wet mounted using 200 µl of 10% glycerol in PBS before imaging. Images were acquired using a ×20/0.75-NA objective on a CyteFinder slide scanning fluorescence instrument (RareCyte). Fluorophores were inactivated by incubating slides in a solution of 4.5% hydrogen peroxide and 24 mM NaOH in PBS and placing under an LED light source for 1 h. To perform CyCIF following Orion imaging, slides were immersed in 1× PBS at 37 °C until the coverslips fell away from the slide. The standard CyCIF method was subsequently performed on these slides.

### IHC

FFPE sections were deparaffinized and dehydrated, and endogenous peroxidase activity was blocked.

Antigen retrieval was performed for 20 min at 100 °C at pH 9 using BOND epitope retrieval solution 2 (Leica Biosystems). Detection was achieved using a Bond Polymer Refine Detection DAB chromogen kit and counterstaining with hematoxylin. Slides were scanned using a RareCyte CyteFinder instrument. Primary antibodies used in IHC are listed in Supplementary Table 2.

### Orion image processing and data analysis

Stitching, channel registration, illumination and geometric distortion correction were performed with Artemis software on the Orion platform, and single-cell data analysis was then performed using MCMICRO modules^30^ including UNMICST2 with cell masks that involved 5-pixel dilation of the nucleus mask. Mean intensity of each channel and morphological features were quantified for each cell.

### Analysis of channel cross-talk

#### Single-plex tonsil images

Tonsil FFPE sections stained with single antibody–ArgoFluor underwent standard acquisition and extraction processing using the Orion instrument. The pixel intensities of all 18 channels from 17 samples were used to quantify bleed through of a given antibody–ArgoFluor complex to the other channels before and after spectral extraction.

#### Eighteen-plex tonsil image

Pearson’s correlation coefficients between all channel pairs were computed using pixel intensities in the 18-plex tonsil image before and after spectral extraction.

### Computational analysis of Orion images and derivation of IFMs

#### IFM computation from Orion data

IFM1 was designed to replicate the logic of the Immunoscore method and was calculated in a semiautomated manner using Orion data. In brief, quantitative data of tumor and immune markers (pan-cytokeratin, CD3ε and CD8α) were gated for high and low cells using a Gaussian mixture model and confirmed by inspection. After gating, the pan-cytokeratin^+^ cells were then used to generate tumor masks using a *k*-nearest neighbor model (kernel size = 25 cells). The tumor margins were derived from tumor masks by expanding 100 µm in either direction from the point of stroma–tumor contact. The CD3^+^ and CD8^+^ fraction, defined as marker-positive cells divided by the total of all successfully segmented cells of all types in either the TC or IM. Tumor and margins were enumerated independently in each sample. The median values of all samples were used as a cutoff to define a subscore as follows: below the median scored as 0 and above the median scored as 1. The final IFM1 value was calculated by adding together the subscores for CD3^+^ and CD8^+^ cells in the TC and IM regions (see Fig. 4b for a flow diagram). The IFM1 score therefore ranged from 0 (CD3^+^ and CD8^+^ low in both regions) to 4 (CD3^+^ and CD8^+^ high in both regions). Similar logic was used to generated other combinations of IFMs. Thirteen selected immune markers (CD3, CD8, CD45, CD45RO, CD68, CD163, CD4, CD20, α-SMA, FOXP3, PD-1 and PD-L1) were gated as described above, and 26 parameters (each marker in the tumor or tumor–stromal interface regions) were generated. The complete combination of 4 of 26 parameters was tested against PFS days for HR. IFM2 was the third best IFM among those combinations, excluding the first and second best combinations, which had some of the same markers as IFM1 (that is, CD3 and CD8); the difference in performance between the top-performing models was insignificant.

#### Leave-one-out test and bootstrapping analysis for IFM2

In the leave-one-out test, the ranks of IFM1 and IFM2 were recalculated with the 40 sets of samples (*N* = 39); each set left out one sample from the original cohort. The collections of ranks from IFM1 and IFM2 were then tested with a pairwise *t*-test. For bootstrapping, the 500 sets of randomly selected samples were used to recalculate the HRs of IFM1 and IFM2 as described above. The collections of HRs from IFM1 and IFM2 were then tested with a pairwise *t*-test. To adjust for multiple hypotheses, the Benjamini–Hochberg procedure was used with a false discovery rate of 0.1.

#### LDA for IFM3 and IFM4

LDA was used to compute spatial neighborhoods as previously described^15^. First, each sample was divided into ‘grids’ of 200 µm (ref. ^2^), and marker frequency was calculated in each grid. The summarized probabilities of all samples were then used to generate the LDA model with 12 topics using collapsed Gibbs sampling in MATLAB. The optimal topic number was determined via varying numbers (between 8 and 16) of topics and evaluating the goodness of fit by calculating the perplexity of a held-out set. After fitting a global LDA model, the individual samples were then applied with the same models to assign topics at the single-cell level.

### CNN to identify IFM3 in H&E images

A publicly available DenseNet161 model^64^ trained with the 100,000 CRC H&E dataset^65^ was used to classify the post-Orion H&E image patches (112 µm^2^) for all the CRC samples. WSI patch prediction was performed with TIAToolbox v1.1.0 (ref. ^64^) on a Windows PC with a Nvidia GeForce GTX 1080 graphics card and batch size of 32. Model performance was reported as *F*_1_ = 0.992. The model has nine output classes: adipose (ADI), background (BACK), debris (DEB), lymphocytes (LYM), mucus (MUC), smooth muscle (MUS), normal colon mucosa (NORM), cancer-associated stroma (STR) and colorectal adenocarcinoma epithelium (TUM). Scripts for reproducing the inference results can be found at https://github.com/labsyspharm/orion-crc)^66^.

The transfer learning of a GoogLeNet model was performed as follows. First, image patches of 224 × 224 pixels were generated from post-Orion H&E images, and the LDA topics were assigned to each patch using Orion data. To exclude low-confidence training data, only patches with more than 20 cells and a dominant topic of >60% were used in the analysis. The selected patches were than separated into training, validation and test sets at a ratio of 0.6:0.2:0.2. The training was done with MATLAB (version 2019b), and the results are shown in Extended Data Fig. 8b. Training parameters are listed in Supplementary Table 5.

### Statistics and reproducibility

Each multimodal image of a tissue section is unique because H&E-stained specimens cannot be reanalyzed by IF. This is a common limitation of high-plex tissue imaging methods in which destructive data collection or tissue damage limits reacquisition of data. Reproducibility of the Orion approach was therefore estimated by performing imaging on serial sections using two instruments in two different locations (Seattle and Boston), as shown in Extended Data Fig. 4, and by comparing Orion with CyCIF images of serial sections as shown in Extended Data Fig. 4. Image analysis assumed multimodal log-normal intensity distributions separable using Gaussian mixture models, but this was not formally tested for all specimens. IFMs were trained on a 40-specimen cohort and tested on an independent of cohort of 34 specimens from different individuals. Specimen sample number was limited by the availability of tissue blocks from the BWH pathology archives. No statistical method was used to predetermine sample size, and no data were excluded from the analyses. The experiments were not randomized, and the investigators were not blinded to allocation during experiments and outcome assessment. These and other limitations of this study are described in the Discussion. Further information on research design is available in the Nature Research Reporting Summary linked to this article.

### Outcome analysis

For all survival analyses, we used a combined survival endpoint of PFS that encompasses both time to disease recurrence for individuals who underwent curative-intent resections (disease-free survival; PFS) and time to progression for individuals with measurable disease (PFS). We used PFS in this paper because it is more familiar. Outcome analysis was performed using Kaplan–Meyer estimation and a log-rank test using the MatSurv function in MATLAB^67^. Cutoffs for IFM1, IFM2 and IFM3 were selected at the median value of the entire cohort, and the cutoff for IFM4 was selected based on IFM1 and IFM3, as described earlier. HRs and confidence intervals were calculated with the log-rank approach: HR = (*O*_a_/*E*_a_)/(*O*_b_/*E*_b_), where *O*_a_ and *O*_b_ are the observed events in each group, and *E*_a_ and *E*_b_ are the number of expected events.

### Reporting summary

Further information on research design is available in the Nature Portfolio Reporting Summary linked to this article.

## Supplementary information

Reporting Summary

Supplementary Tables Supplementary Tables 1–5.

## Data Availability

All images and derived data are available without restriction via the NCI Human Tumor Atlas Network Portal (https://humantumoratlas.org/explore) in accordance with NCI Moonshot Policies. The Human Tumor Atlas Network participant (specimen) ID numbers are listed in Supplementary Table 3. All other data supporting the findings of this study are available via an index page on GitHub that has been archived on Zenodo (10.5281/zenodo.7637655) (ref. ^66^). Source data are provided with this paper.

## Source data

Source Data Fig. 1 Intensities of fluorochromes in each Orion channel before and after spectral extraction.
Source Data Fig. 2 Plots of the fraction of cells positive for the indicated markers from whole-slide Orion IF and CyCIF images acquired from neighboring sections from 29 FFPE CRC specimens.
Source Data Fig. 3 Fraction of the total number of ‘unidentifiable’ cells assigned with the CNN model.
Source Data Fig. 4 IFM1 and IFM2 versus PFS days. These data are identical to the data for Fig. 5.
Source Data Fig. 5 IFM1 and IFM2 versus PFS days. These data are identical to the data for Fig. 4.
Source Data Fig. 6 Fractions of topics in each sample, the relationship to PFS days and the markers per topic.
Source Data Fig. 7 IFM3 and IFM4 versus PFS days.
Source Data Extended Data Fig. 1 Emission spectra of different ArgoFluors in **b**.
Source Data Extended Data Fig. 2 Mean pixel-to-pixel correlation matrix of channel pairs in **d**.
Source Data Extended Data Fig. 3 Plots of the fraction of cells positive for the indicated markers from whole-slide Orion IF and CyCIF images acquired from neighboring sections from 29 FFPE CRC specimens. These data are identical to the data for Fig. 2.
Source Data Extended Data Fig. 4 Evaluation of Orion data collected at two performance sites.
Source Data Extended Data Fig. 6 Fractions of marker-positive cells versus PFS days.
Source Data Extended Data Fig. 7 Relationship of PFS days per topic. These data are identical to the data for Fig. 6d.
Source Data Extended Data Fig. 8 Generated using the same inputs as Fig. 7a,b. These data are identical to the data for Fig. 7.

## Code availability

Image and data analyses were performed using customized scripts in Python, ImageJ and MATLAB. All code is available under an MIT open-source license via an index page on GitHub that has been archived on Zenodo (10.5281/zenodo.7637655) (ref. ^66^).
