# Supplementary material for: High-plex immunofluorescence imaging and traditional histology of the same tissue section for discovering image-based biomarkers
Source: Nat Cancer. 2023 Jun 22;4(7):1036–52. doi: 10.1038/s43018-023-00576-1 (PMC10368530; doi:10.1038/s43018-023-00576-1)
Supplement: Supplementary file 1 [file 43018_2023_576_MOESM1_ESM.pdf]

nature portfolio

Corresponding author(s): Sandro Santagata and Peter K. Sorger

Last updated by author(s): April 25, 2023

Reporting Summary

Nature Portfolio wishes to improve the reproducibility of the work that we publish. This form provides structure for consistency and transparency in reporting. For further information on Nature Portfolio policies, see our Editorial Policies and the Editorial Policy Checklist.

Statistics

For all statistical analyses, confirm that the following items are present in the figure legend, table legend, main text, or Methods section.

n/a	Confirmed	
☐	☒	The exact sample size (*n*) for each experimental group/condition, given as a discrete number and unit of measurement
☐	☒	A statement on whether measurements were taken from distinct samples or whether the same sample was measured repeatedly
☐	☒	The statistical test(s) used AND whether they are one- or two-sided<br>*Only common tests should be described solely by name; describe more complex techniques in the Methods section.*
☐	☒	A description of all covariates tested
☐	☒	A description of any assumptions or corrections, such as tests of normality and adjustment for multiple comparisons
☐	☒	A full description of the statistical parameters including central tendency (e.g. means) or other basic estimates (e.g. regression coefficient) AND variation (e.g. standard deviation) or associated estimates of uncertainty (e.g. confidence intervals)
☐	☒	For null hypothesis testing, the test statistic (e.g. *F*, *t*, *r*) with confidence intervals, effect sizes, degrees of freedom and *P* value noted<br>*Give P values as exact values whenever suitable.*
☒	☐	For Bayesian analysis, information on the choice of priors and Markov chain Monte Carlo settings
☒	☐	For hierarchical and complex designs, identification of the appropriate level for tests and full reporting of outcomes
☐	☒	Estimates of effect sizes (e.g. Cohen's *d*, Pearson's *r*), indicating how they were calculated

Our web collection on statistics for biologists contains articles on many of the points above.

Software and code

Policy information about availability of computer code

Data collection	Data supporting the findings of this study are available via an index page (https://github.com/labsyspharm/orion-crc/blob/main/datarelease-README.md) on GitHub that has been archived on Zenodo (https://zenodo.org/) - https://doi.org/10.5281/zenodo.7637655.
Data analysis	Image and data analysis was performed using customized scripts in Python, ImageJ, and MATLAB. All code is available under an MIT open-source license via an index page on GitHub (https://github.com/labsyspharm/orion-crc) that has been archived on Zenodo (https://zenodo.org/) - https://doi.org/10.5281/zenodo.7637655

For manuscripts utilizing custom algorithms or software that are central to the research but not yet described in published literature, software must be made available to editors and reviewers. We strongly encourage code deposition in a community repository (e.g. GitHub). See the Nature Portfolio guidelines for submitting code & software for further information.

Data

Policy information about availability of data

All manuscripts must include a data availability statement. This statement should provide the following information, where applicable:

- Accession codes, unique identifiers, or web links for publicly available datasets
- A description of any restrictions on data availability
- For clinical datasets or third party data, please ensure that the statement adheres to our policy

All image and derived data are available without restriction via the NCI Human Tumor Atlas Network (HTAN) Portal (https://humantumoratlas.org/explore) in

accordance with NCI Moonshot Policies. HTAN participant (specimen) ID is listed in Extended Data Table 3. All other data supporting the findings of this study are available via an index page on GitHub that has been archived on Zenodo (https://zenodo.org/) - https://doi.org/10.5281/zenodo.7637655. Source data have been provided as Source Data files.

Human research participants

Policy information about studies involving human research participants and Sex and Gender in Research.

Reporting on sex and gender	Two cohorts from same biobank were assembled, the first with 40 patients with state II-IV CRC, then the second with 34 patients with no consideration for the sex of the participants. All samples were collected at the time of initial diagnosis.
Population characteristics	In the current work, specific limitations include a relatively small cohort size, the absence of pre-registration, and the acquisition of specimens from a single institution. As a result we do not fully control for all relevant covariates (e.g., depth of invasion, sex, age, race, clinical stage, etc.) and more progressors were included in our cohort than would be observed in an unselected population, biasing the cohort to more serious disease (~50% two-year disease-free survival for Stage III colon cancer in our cohort v. an accepted value of ~80%). The cohort included 39 females and 35 male. See Supplementary Table 3.
Recruitment	Samples were obtained from the archives of Brigham and Womens under a discarded tissue protocol.
Ethics oversight	The research described in this manuscript complies with all relevant ethical regulations and was reviewed and approved by the Institutional Review Boards (IRB) at Brigham and Women's Hospital (BWH), Harvard Medical School (HMS), and Dana Farber Cancer Institute (DFCI). Formaldehyde-fixed and paraffin-embedded (FFPE) tissue samples were used after diagnosis and informed written patient consent under Dana-Farber Cancer Institute IRB protocol 17-000.

Note that full information on the approval of the study protocol must also be provided in the manuscript.

Field-specific reporting

Please select the one below that is the best fit for your research. If you are not sure, read the appropriate sections before making your selection.

☒ Life sciences ☐ Behavioural & social sciences ☐ Ecological, evolutionary & environmental sciences

For a reference copy of the document with all sections, see nature.com/documents/nr-reporting-summary-flat.pdf

Life sciences study design

All studies must disclose on these points even when the disclosure is negative.

Sample size	74 samples were chosen. These represent the largest number of samples that could be practically analyzed within the time and financial constraints. See Limitations section.
Data exclusions	For one patient, the specimen was available but there was insufficient clinical follow-up for progression.
Replication	6 samples were selected to perform the reproducibility test. The replicated samples were stained and scanned in different sites, then results were presented in the extended data figure 4.
Randomization	Model features were evaluated using leave-one-out cross validation and bootstrapping.
Blinding	Conventional blinding is not relevant to this type of retrospective non-interventional study. Details of the model development are described in detail in the text and methods.

Reporting for specific materials, systems and methods

We require information from authors about some types of materials, experimental systems and methods used in many studies. Here, indicate whether each material, system or method listed is relevant to your study. If you are not sure if a list item applies to your research, read the appropriate section before selecting a response.

Materials & experimental systems

n/a	Involved in the study
☐	☒ Antibodies
☒	☐ Eukaryotic cell lines
☒	☐ Palaeontology and archaeology
☒	☐ Animals and other organisms
☒	☐ Clinical data
☒	☐ Dual use research of concern

Methods

n/a	Involved in the study
☒	☐ ChIP-seq
☒	☐ Flow cytometry
☒	☐ MRI-based neuroimaging

Antibodies

Antibodies used

Clone information and RRID of primary and secondary antibodies used in the manuscript can be found in Supplementary Table 2.

Target,Clone,Antibody RRID,Concentration
CD31,EPR3094,"RareCyte Cat# 52-1005-501, RRID:AB_2922783",2 µg/mL
CD45,D9M8I,"RareCyte Cat# AFAB-010-B, RRID:AB_2922785",1 µg/mL
CD68,KP1 + C68/684,"RareCyte Cat# 52-1008-501, RRID:AB_2922786",0.5 µg/mL
CD4,NIUG0,"RareCyte Cat# 52-1002-501, RRID:AB_2922787",2 µg/mL
FOXP3,236A/E7,"RareCyte Cat# AFAB-018-B, RRID:AB_2922788",2 µg/mL
CD8a,AMC908,"RareCyte Cat# 52-1003-601, RRID:AB_2922789",0.5 µg/mL
CD45RO,UCHL1,"RareCyte Cat# 52-1007-601, RRID:AB_2922790",2 µg/mL
CD20,L26,"RareCyte Cat# 52-1004-601, RRID:AB_2922791",1 µg/mL
PD-L1,E1L3N,"RareCyte Cat# 52-1018-601, RRID:AB_2922792",2 µg/mL
CD3e,D7A6E,"RareCyte Cat# 52-1001-601, RRID:AB_2922793",4 µg/mL
CD163,EPR14643,"RareCyte Cat# AFAB-014-B, RRID:AB_2922794",1 µg/mL
E-Cadherin,4A2,"RareCyte Cat# 52-1010-701, RRID:AB_2922795",2 µg/mL
PD-1,EPR4877(2) ,"RareCyte Cat# 52-1017-701, RRID:AB_2922797",1 µg/mL
Ki67,D3B5,"RareCyte Cat# AFAB-021-B, RRID:AB_2922798",2 µg/mL
Pan-CK (DIG),AE1/AE3/C11,"RareCyte Cat# 52-1015-801, RRID:AB_2922799",5 µg/mL
SMA (BIO),1A4,"RareCyte Cat# 52-1023-111, RRID:AB_2922801",2 µg/mL

Validation

Each ArgoFluor™ conjugated primary antibody was validated by comparing single-plex Immunofluorescence (IF) staining patterns to the patterns obtained by gold-standard indirect Immunohistochemistry (IHC) on tissue sections known to express the antibody targets.
